# miR-126-3p down-regulation contributes to dabrafenib acquired resistance in melanoma by up-regulating ADAM9 and VEGF-A

**DOI:** 10.1186/s13046-019-1238-4

**Published:** 2019-06-21

**Authors:** Simona Caporali, Adriana Amaro, Lauretta Levati, Ester Alvino, Pedro Miguel Lacal, Simona Mastroeni, Federica Ruffini, Laura Bonmassar, Gian Carlo Antonini Cappellini, Nadia Felli, Alessandra Carè, Ulrich Pfeffer, Stefania D’Atri

**Affiliations:** 1 Laboratory of Molecular Oncology, Istituto Dermopatico dell’Immacolata, IDI-IRCCS, Via dei Monti di Creta 104, 00167 Rome, Italy; 2 Molecular Pathology, IRCCS-Ospedale Policlinico San Martino, Genoa, Italy; 3 Institute of Translational Pharmacology, National Council of Research, Rome, Italy; 4 Clinical Epidemiology Unit, IDI-IRCCS, Rome, Italy; 5 Department of Oncology and Dermatological Oncology, IDI-IRCCS, Rome, Italy; 6 Department of Oncology and Molecular Medicine, Istituto Superiore di Sanità, Rome, Italy; 7 Center of Gender Medicine, Istituto Superiore di Sanità, Rome, Italy

**Keywords:** miR-126-3p, melanoma, BRAF inhibitors, acquired resistance, proliferation, invasiveness

## Abstract

**Background:**

Development of resistance to inhibitors of BRAF (BRAFi) and MEK (MEKi) remains a great challenge for targeted therapy in patients with BRAF-mutant melanoma. Here, we explored the role of miRNAs in melanoma acquired resistance to BRAFi.

**Methods:**

miRNA expression in two BRAF-mutant melanoma cell lines and their dabrafenib-resistant sublines was determined using Affymetrix GeneChip® miRNA 3.1 microarrays and/or qRT-PCR. The effects of miR-126-3p re-expression on proliferation, apoptosis, cell cycle, ERK1/2 and AKT phosphorylation, dabrafenib sensitivity, invasiveness and VEGF-A secretion were evaluated in the dabrafenib-resistant sublines using MTT assays, flow cytometry, immunoblotting, invasion assays in Boyden chambers and ELISA. ADAM9, PIK3R2, MMP7 and CXCR4 expression in the sensitive and dabrafenib-resistant cells was determined by immunoblotting. Small RNA interference was performed to investigate the consequence of *VEGFA* or *ADAM9* silencing on proliferation, invasiveness or dabrafenib sensitivity of the resistant sublines. Long-term proliferation assays were carried out in dabrafenib-sensitive cells to assess the effects of enforced miR-126-3p expression or *ADAM9* silencing on resistance development. VEGF-A serum levels in melanoma patients treated with BRAFi or BRAFi+MEKi were evaluated at baseline (T0), after two months of treatment (T2) and at progression (TP) by ELISA.

**Results:**

miR-126-3p was significantly down-regulated in the dabrafenib-resistant sublines as compared with their parental counterparts. miR-126-3p replacement in the drug-resistant cells inhibited proliferation, cell cycle progression, phosphorylation of ERK1/2 and/or AKT, invasiveness, VEGF-A and ADAM9 expression, and increased dabrafenib sensitivity. *VEGFA* or *ADAM9* silencing impaired proliferation and invasiveness of the drug-resistant sublines. *ADAM9* knock-down in the resistant cells increased dabrafenib sensitivity, whereas miR-126-3p enforced expression or ADAM9 silencing in the drug-sensitive cells delayed the development of resistance. At T0 and T2, statistically significant differences were observed in VEGF-A serum levels between patients who responded to therapy and patients who did not. In responder patients, a significant increase of VEGF-A levels was observed at TP *versus* T2.

**Conclusions:**

Strategies restoring miR-126-3p expression or targeting VEGF-A or ADAM9 could restrain growth and metastasis of dabrafenib-resistant melanomas and increase their drug sensitivity. Circulating VEGF-A is a promising biomarker for predicting patients’ response to BRAFi or BRAFi+MEKi and for monitoring the onset of resistance.

**Electronic supplementary material:**

The online version of this article (10.1186/s13046-019-1238-4) contains supplementary material, which is available to authorized users.

## Background

Melanoma is the most deadly type of skin cancer. Although early-stage melanoma is often curable by surgical resection, advanced melanoma has a dismal prognosis, and until recent years limited and palliative treatment options were available for its management [1, 2].

Between 2011 and 2018, the BRAF inhibitors (BRAFi) vemurafenib and dabrafenib, the MEK inhibitor (MEKi) trametinib and combinations of BRAFi and MEKi (dabrafenib+trametinib, vemurafenib+cobimetinib, encorafenib+binimetinib) have been approved as first line therapies for patients with unresectable or metastatic melanoma harboring BRAF^V600E^ (vemurafenib, dabrafenib) or BRAF^V600E/K^ (trametinib, combined therapies). Objective response rates of 50-60% and of 65-75% are obtained with BRAFi and BRAFi+MEKi therapy, respectively, with a significant increase of progression-free and overall survival [3, 4]. However, despite most patients experience an impressive initial response to BRAFi and to the combination of BRAFi+MEKi, the development of drug resistance limits the long-term efficacy of therapy in nearly all patients [5–7]. Moreover, a non-negligible percentage of patients shows primary resistance.

So far, a variety of molecular mechanisms underlying primary and/or acquired resistance to BRAFi and MEKi has been described, including mutations in *NRAS*, *MAP2K1* or *MAP2K2*, *NF1* loss, *BRAF*^*V600E*^ amplification or splice variant expression, mutations in members of the PI3K/AKT/mTOR signaling axis, and over-expression and/or activation of receptor tyrosine kinases (RTKs) [5–7]. Of note, in a considerable percentage of BRAFi-resistant tumor samples analyzed in genomic studies [8, 9] no validated mutational mechanisms of resistance have been detected. Therefore, the identification of epigenetic and/or post-transcriptional alterations involved in resistance to BRAFi appears of relevance for developing new combinatorial therapeutic approaches aimed at increasing drug sensitivity and overcoming the emergence of secondary resistance.

MicroRNAs (miRNAs), small non-coding RNAs of ~19–25 nucleotides, modulate gene expression through imperfect base-pairing with specific sequences in the 3′-untranslated regions of target mRNAs, thereby inducing transcript destabilization, translational inhibition, or both [10]. A single miRNA can regulate the expression of hundreds of genes, and the expression of a single gene can be regulated by multiple miRNAs. Moreover, one miRNA often targets multiple genes that are involved in a specific signaling cascade, thus making miRNAs potent regulators of most cellular processes, including apoptosis, proliferation, differentiation, and metabolism [10]. miRNAs are also important modulators of immune response in both physiological and pathological conditions [11].

Aberrant expression of miRNAs has been demonstrated in a variety of human cancers, including melanoma [12–15]. In this context, miRNAs can operate as oncogenes or tumor suppressor genes. Moreover, miRNAs have been shown to have both diagnostic and prognostic significance and to potentially constitute novel targets and therapeutic agents for cancer treatment [12–15]. Experimental evidence also indicates that miRNAs are involved in tumor primary and secondary resistance to various anticancer agents [16, 17]. Nevertheless, only a limited number of studies have explored the role of miRNA alterations in melanoma acquired resistance to BRAFi. In this regard, Liu et al [18] demonstrated that down-regulation of miR-200c occurred in melanoma cell lines, specimens and patient-derived xenografts characterized by acquired resistance to BRAFi and that replacement of this miRNA, or silencing of its target Bim, in the BRAFi-resistant cell lines increased sensitivity to the BRAFi PLX4720. In a different set of melanoma cell lines, Sun et al [19] found that acquired resistance to vemurafenib was associated with a strong down-regulation of miR-7 and up-regulation of its targets epidermal growth factor receptor (EGFR), insulin-like growth factor-1 receptor (IGF-1R) and CRAF. Moreover, the authors showed that re-expression of miR-7 impaired proliferation of the drug-resistant cell lines both in vitro and in vivo in a murine model, most likely through combined inhibition of EGFR, IGF-1R and CRAF. A decrease in the expression of miR-579-3p has also been reported to be involved in the development of drug resistance in melanoma cells treated in vitro with vemurafenib, and in patients subjected to BRAFi therapy [20]. Enforced expression of miR-579-3p not only sensitized the resistant cells to vemurafenib, but also impaired the establishment of resistance in sensitive cells chronically exposed to the drug. More recently, Fattore et al [21] identified down-regulation of miR-204-5p and miR-199b-5p in BRAFi-resistant melanoma cell lines and specimens, and provided strong experimental evidence of their involvement in the regulation of cell proliferation, apoptosis and sensitivity to BRAFi. Up-regulation of selected miRNAs has also been implicated in acquired resistance of melanoma cells to BRAFi. In particular, increased expression of miR-34, miR-100, miR-125b [22], miR-1246 [23], miR-125a [24], miR-204-5p and miR-211-5p [25], miR-4443 and miR-4488 [21] has been described.

In this study, we showed that changes in miRNA expression profile were associated with acquired resistance of melanoma cells to dabrafenib, and that miR-126-3p, known to be involved in melanoma progression and invasiveness [26], was among the most down-regulated miRNAs. Re-establishment of miR-126-3p expression or silencing of its target genes *VEGFA* or *ADAM*9 impaired proliferation and invasiveness of dabrafenib-resistant cells. Moreover, in those cells, miR-126-3p replacement as well as *ADAM9* knock-down increased sensitivity to the BRAFi or counteracted its growth stimulating effect. Notably, enforced expression of miR-126-3p or *ADAM9* silencing in drug-sensitive cells delayed the development of resistance upon long-term exposure to dabrafenib. Finally, we found that high serum levels of VEGF-A were associated with poor clinical response in melanoma patients treated with BRAFi or BRAFi+MEKi. Replacement of miR-126-3p or inhibition of VEGF-A or ADAM9 expression may be, therefore, novel therapeutic approaches to increase the efficacy of BRAFi. Moreover, circulating VEGF-A may be a useful biomarker to improve patient selection for targeted therapy and for monitoring the onset of resistance.

## Materials and methods

### Cell cultures

The BRAF-mutant human melanoma cell lines A375 and SK-Mel28 were purchased from the European Collection of Cell Cultures (Salisbury, UK) and American Type Culture Collection (ATCC, Manassas, VA), respectively. The cells were cultured in BioWhittaker™ RPMI-1640 medium (LONZA, Verviers, Belgium) supplemented with 10% fetal bovine serum (Sigma-Aldrich, St. Louis, MO), 2 mM BioWhittaker™ L-glutamine (LONZA), and 50 μg/ml BioWhittaker™ gentamicin (LONZA) (hereafter referred to as complete medium, CM).

The dabrafenib-resistant A375R and SK-Mel28R cell lines were generated in our laboratory by growing A375 and SK-Mel28 cells in gradually increasing concentrations of the drug (up to 1.5 μM) [27, 28]. A375R and SK-Mel28R cell lines were maintained in CM supplemented with 1.5 μM dabrafenib. The two pair of sensitive/resistant cell lines were authenticated by STR profiling (BMR genomics, Padova, Italy).

### Drugs, chemicals and antibodies

Dabrafenib was purchased from Selleckchem (Houston, TX) and dissolved in dimethyl sulfoxide (DMSO) (Sigma-Aldrich) at a final concentration of 1.92 mM. Dabrafenib was stored as stock solutions at -80°C and diluted in CM just before use. 3-(4,5-dimethylthiazol-2-yl)-2,5-diphenyltetrazolium bromide (MTT) was purchased from Sigma-Aldrich, dissolved at a concentration of 5 mg/ml in GIBCO™ Phosphate-Buffered Saline (PBS) (Invitrogen, Thermo Fisher Scientific, Waltham, MA) and stored at 4°C. Mouse monoclonal antibodies (mAb) against ADAM9 (sc-377233), and rabbit polyclonal antibody against human β-tubulin (sc-9104) were purchased from Santa Cruz Biotechnology, Inc. (Santa Cruz, CA). mAb against PIK3R2 (#05-217) was purchased from Upstate Biotechnology (Lake Placid, NY). mAb against MMP7 (#111433) was purchased from R&D Systems (Minneapolis, MN). Rabbit polyclonal antibody against CXCR4 (ab2074) was purchased from Abcam (Cambridge, MA). Rabbit polyclonal antibodies against ERK1/2 (#9102), phospho-ERK1/2 (Thr202/Tyr204) (#9101), AKT (#9272) and phospho-AKT (Ser473) (#9271) were purchased from Cell Signaling Technology, Inc. (Beverly, MA). Reagents for sodium dodecyl sulphate (SDS)-polyacrylamide gel electrophoresis were all purchased from Bio-Rad Laboratories, Inc. (Hercules, CA).

### miRNA expression profiling

For miRNA expression profiling, A375 and A375R cells were removed from culture, seeded into BD Falcon™ tissue culture dishes (BD Biosciences, Bedford, MA), and maintained in CM for 48 h. The cells were then detached with a solution of 0.5 mM EDTA in PBS, washed in PBS and frozen in a RNA-*later*®/PBS solution. Total RNA was isolated using the mirVana™ miRNA Isolation Kit (Ambion, Grand Island, NY) following the manufacturer's protocol. RNA was quantified using the NanoDrop ND-1000 spectrophotometer (Thermo Fisher Scientific) and checked for integrity and quality by 2100 Bioanalyzer (Agilent Genomics, Santa Clara, CA) before microarray hybridization. One μg of RNA was biotin labeled using the FlashTag™ HSR RNA labeling kit for Affymetrix GeneChip™ miRNA 3.1 Array Strip arrays (Affymetrix Inc., Santa Clara, CA) and then 100 ng were hybridized to GeneChip™ miRNA 3.1 Array Strip, according to manufacturer's instructions. The array strips were washed and thereafter scanned with an Affymetrix GeneAtlas® Scanner, controlled by Transcriptome Analysis Console software, to produce CEL intensity files. Three biological replicates were set up for each sample. Annotations were obtained from Thermo Fisher website. CEL file for each array was imported into R/BioConductor and pre-processed using RMA (Robust Multichip Averaging) with quantile normalization.

Statistically significant expression changes were determined using permutation tests (Significance Analysis of Microarrays, SAM) (http://statweb.stanford.edu/~tibs/SAM/) [29] and visualized using hierarchical clustering with Pearson correlation and average linkage, performed using MeV 4.0 software (http://mev.tm4.org/) [30]. miRNAs regulated at least 2-fold in A375R cells in comparison to A375 cells, with a false discovery rate (FDR) of 0%, were considered. Potential target genes of differentially expressed miRNAs as predicted by TargetScan [31] were obtained using miRWalk 2.0 (http://zmf.umm.uni-heidelberg.de/apps/zmf/mirwalk2/) [32]. Enrichment analysis of KEGG pathways annotations of target genes of differentially expressed miRNAs was performed using the EnrichR online tool (http://amp.pharm.mssm.edu/Enrichr/) [33]. The data were deposited in the Gene Expression Omnibus repository (accession number: GSE117666).

### Real-time quantitative RT-PCR (qRT-PCR) analysis.

The expression of mature hsa-miR-126-3p, hsa-miR-146a-5p, hsa-miR-424-3p, hsa-miR-503-5p and hsa-miR-509-3p, was assayed using the TaqMan® MicroRNA Reverse Transcription Kit, the TaqMan® Universal PCR Master Mix No AmpErase® UNG, and the specific TaqMan® MicroRNA Assays, all purchased from Applied Biosystems (Foster City, CA). All experimental procedures were performed according to the manufacturer’s protocols. Ten ng of RNA were reverse transcribed in a final volume of 15 μl, and qRT-PCR was done on an ABI PRISM 7000 Sequence Detection System (Applied Biosystems) in a final volume of 20 μl. All qRT-PCR reactions were run in duplicate. The expression of miRNAs relative to RNU44 was determined using the formula 2^-ΔCt^, where ΔC_T_ = C_TmiR_- C_TRNU44_, and C_T_ (i.e. threshold cycle) indicates the fractional cycle number at which the amount of amplified target reaches a fixed threshold. To simplify data presentation, the relative expression values were multiplied by 10^5^_._

### Transient transfection and drug treatment for functional assays

Transfection was performed using Lipofectamine™ RNAiMAX reagent (Invitrogen Corporation, Carlsbad, CA) according to the manufacturer’s protocol.

For proliferation assays, melanoma cells were suspended in CM without antibiotics, seeded into BD Falcon™ 96-well plates and allowed to adhere at 37°C in a 5% CO_2_ atmosphere for 18 h. The cells were then transfected with 50 nM of Ambion® Pre-miR™ miRNA precursor (hereafter referred to as pre-miR) of hsa-miR-126-3p or hsa-miR-146a-5p, or with *mir*Vana™ miRNA inhibitor (hereafter referred to as anti-miR) of hsa-miR-424-3p, hsa-miR-503-5p or hsa-miR-509-3p or with pre-miR miRNA precursor or anti-miR miRNA inhibitor negative controls, (hereafter referred to as pre-miR-CTRL and anti-miR-CTRL, respectively) all purchased from Ambion. Six days after transfection, the cells were analyzed for proliferation using the MTT assay, as previously described [34]. Three replica wells were used for each group.

For chemosensitivity assays, melanoma cells were suspended in CM without antibiotics, plated and allowed to adhere as described above, and then transfected with 50 nM pre-miR-126-3p or pre-miR-CTRL or with 10 nM (SK-Mel28R) or 50 nM (A375R) of a pool of two small interfering RNAs directed against *ADAM9* (siADAM9; sc-41408, Santa Cruz Biotechnology, Inc) or AllStars Negative Control siRNA (siCTRL; #1027281, Qiagen, Hilden, Germany). After 24 h of incubation, the cells were exposed to DMSO alone or to increasing concentrations of dabrafenib. The plates were incubated at 37°C for five days and cell growth was then evaluated by the MTT assay. Three replica wells were used for each group.

For long-term proliferation assays, SK-Mel28 cells were plated in triplicate into three BD Falcon™ 96-well plates, allowed to adhere at 37°C for 18 h, and then transfected with 50 nM pre-miR-126-3p or pre-miR-CTRL (day 0). After 24 h of culture (day 1), 100 nM dabrafenib or DMSO was added to the wells. On day 8, the cells of one plate were fixed with ethanol, stained with 0.5% crystal violet and photographed. Thereafter, for quantitative analysis of proliferation, crystal violet was solubilized with 10% acid acetic and absorbance was read at 595 nm. In the remaining plates, culture medium was changed and the cells were subjected to a new cycle of transfection and drug treatment as described above. This procedure was repeated every eight days, and cell growth evaluated on day 56 and on day 96. In a different set of experiments, A375 and SK-Mel28 cells were plated as described above, transfected with 10 nM (SK-Mel28) or 50 nM (A375) siADAM9 or siCTRL (day 0), and treated with 100 nM dabrafenib or with DMSO (day 1). On day 8, the cells of one plate were photographed and processed to evaluate proliferation, whereas the cells of the remaining plates were transfected and treated with dabrafenib every eight days. Cell proliferation was assessed on day 16 and day 24 (A375) or on day 24 and day 32 (SK-Mel28).

For invasion assays, melanoma cells were suspended in CM without antibiotics, seeded into BD Falcon™ tissue culture dishes allowed to adhere at 37°C for 18 h, and then transfected with 50 nM of pre-miR-126-3p or pre-miR-CTRL, or with 50 nM of an anti-VEGF-A siRNA (siVEGFA; sc-29520, Santa Cruz Biotechnology, Inc.) or with 10 nM (SK-Mel28R) or 50 nM (A375R) siADAM9 or siCTRL. The cells transfected with pre-miR-126-3p or pre-miR-CTRL were exposed to 100 nM dabrafenib or DMSO 24 h after transfection and 48 h later assessed for invasiveness, whereas the cells transfected with siVEGFA or siADAM9 were tested for invasiveness 72 h after transfection.

For evaluation of VEGF-A secretion, melanoma cells were suspended in CM without antibiotics, plated, allowed to adhere as described for invasion assays, and then transfected with 50 nM of pre-miR-126-3p or pre-miR-CTRL, or with 50 nM siVEGFA or siCTRL. After 72 h of incubation, culture supernatants were recovered for VEGF-A determination by ELISA. The cells were detached and counted for normalization of VEGF-A secretion and, in the case of siCTRL- and siVEGFA-transfected cells, also for assessment of proliferation.

For Western blot analysis, melanoma cells were suspended in CM without antibiotics, seeded into BD Falcon™ tissue culture dishes and 18 h later transfected with 50 nM pre-miR-126-3p or pre-miR-CTRL, or with 10 nM (SK-Mel28R) or 50 nM (A375R) siADAM9 or siCTRL. Forty-eight or 72 h after transfection the cells were recovered for protein extract preparation.

For evaluation of cell cycle perturbations and apoposis, melanoma cells were seeded and transfected with 50 nM pre-miR-126-3p or pre-miR-CTRL as described above and recovered for flow cytometry analysis 72 h after transfection.

### Invasion assay in Boyden chambers

This assay was performed as previously described [27, 28]. Briefly, melanoma cells were removed from culture, washed, suspended in invasion medium (1 μg/ml heparin/0.1% bovine serum albumin in RPMI-1640) and loaded (2x10^5^ cells) into the upper compartment of Boyden chambers equipped with 8-μm pore diameter polycarbonate filters (Nuclepore, Whatman Inc., Clifton, NJ) coated with 20 μg of BD Matrigel™ Basement Membrane Matrix (BD Biosciences). Invasion medium was added to the lower compartment of the chambers. For each experimental condition, three Boyden chambers were set up. After incubation of the Boyden chambers at 37°C in a 5% CO_2_ atmosphere for 4 h, the filters were removed from the chambers and the cells were fixed in ethanol for 5 min and stained in 0.5% crystal violet for 15 min. The cells from the upper surface of the filter were removed by wiping with a cotton swab and the migrated cells, attached to the lower surface of the filters, were counted under the microscope. Twelve microscopic fields (x200 magnification), randomly selected on triplicate filters, were scored for each experimental condition.

### Cell cycle and apoptosis analysis

Adherent and floating cells were collected from cultures, washed in PBS and fixed with 70% ethanol at -20°C for 18 h. The cells were then centrifuged, resuspended in 1 ml of hypotonic solution containing 50 μg/ml propidium iodide, 0.1% sodium citrate, 0.1% Triton-X, and 10 μg/ml RNase, and incubated in the dark, at room temperature for 30 min. Data collection was gated utilizing forward light scatter and side light scatter to exclude cell debris and cell aggregates. The propidium iodide fluorescence was measured on a linear scale using a FACSCalibur flow cytometer (BD Biosciences). Apoptotic cells were determined by their hypochromic, sub-G_1_ staining profiles, whereas the percentage of cells in the different phases of cell cycle were determined using the Cyflogic software (http://www.cyflogic.com/) after excluding sub-G1 cells.

### Western blot analysis

Melanoma cells were recovered from culture, washed and total cellular extracts were prepared as described previously [34]. Fifteen μg of proteins per sample were run on 8% SDS-polyacrilamide gels, transferred to nitrocellulose membranes (Amersham Biosciences, Buckinghamshire, UK) and blocked with 5% non-fat milk in Tris-buffered saline supplemented with 0.1% Tween 20 (TBST) for 1 h at room temperature. The membranes were then incubated in TBST containing 5% non-fat milk overnight at 4°C with primary antibodies at the following dilutions: anti-β-tubulin 1:1000, anti-ADAM9 1:500, anti-PIK3R2 1:1000, anti-CXCR4 1:500 and anti-MMP7 1:2500, anti-ERK1/2 1:500, anti-phospho-ERK1/2 (Thr202/Tyr204) 1:500, anti-AKT 1:500, anti-phospho-AKT (Ser473) 1:500. The anti-β-tubulin antibody was used as an internal standard for loading. Immunodetection was carried out using appropriate horseradish peroxidase-linked secondary antibodies and developed with Clarity™ Western ECL Substrate (Bio-Rad Laboratories, Inc.).

### Evaluation of VEGF-A secretion

Culture supernatants were collected, centrifuged at 600 x g for 10 min to remove cells in suspension and debris, and frozen at -20°C until use. Cells were detached with a solution of 1.5 mM EDTA in PBS and counted to determine the total number of cells in the culture. Quantification of VEGF-A amount in the culture supernatants was performed as previously described [35]. The amount of VEGF-A was normalized to the number of total cells counted at the time of supernatant collection.

### 3’UTR luciferase assay

The empty pMirTarget Vector (pMirE), the recombinant vectors containing wild-type (pMirVEGF-wt, #SC217124) or mutant (pMirVEGF-mut, #CW303621, custom designed) VEGF-A mRNA 3′UTR, were purchased from Origene Technologies, Inc. (Rockville, MD). The mutant seed sequence of miR-126-3p (5’...**A***TC***G***AT***C** … 3’) was previously validated [36]. The *Renilla* luciferase expression vector pRL-null was purchased from Promega Corporation (Madison, WI). A375R cells were seeded into BD Falcon™ 24-well plates and allowed to adhere at 37°C for 18 h. The cells were then co-transfected with 100 ng of the desired vector and 10 ng of pRL-null vector along with 50 ng of pre-miR-126-3p or pre-miR-CTRL, creating six test groups. Luciferase activity was determined 48 h after co-transfection using the Dual-Luciferase® Reporter Assay (Promega). Firefly luciferase activity was normalized to *Renilla* luciferase activity for each sample.

### Patients

Serum levels of VEGF-A were determined in 26 patients with BRAF^V600^-mutant metastatic cutaneous melanoma consecutively enrolled for treatment with either dabrafenib, vemurafenib, dabrafenib plus trametinib or vemurafenib plus cobimetinib at Istituto Dermopatico dell’Immacolata, IDI-IRCCS and from whom peripheral blood samples had been sequentially collected before therapy commencement and up to disease progression. Baseline evaluation included medical history, physical examination, and radiologic tumor assessment with computer tomography (CT) or positron emission tomography scans. Dabrafenib (Tafinlar®) was given at the dose of 150 mg BID, vemurafenib (Zelboraf®) at the dose of 960 mg BID, dabrafenib plus trametinib (Mekinist®) at the dose of 150 mg BID and 2 mg/die, respectively, and vemurafenib plus cobimetinib at the dose of 960 mg BID and 60 mg/die, respectively, for three weeks with one week of break. All patients underwent physical examination and assessment of biochemical parameters monthly, whereas tumor response was determined with CT every three months. Tumor response was classified as complete response (CR), partial response (PR), stable disease (SD) or progressive disease (PD) according to RECIST 1.1 criteria. Time to treatment failure (TTF) was defined as the time from the start of therapy to the first observation of disease progression per RECIST 1.1. The study was conducted in accordance with Good Clinical Practice Guidelines and the Declaration of Helsinki. The study was also approved by the IDI-IRCCS Ethics Committee (ID #407/1, 2013 and #407/2, 2016) and a written informed consent was obtained from all patients.

### Serum preparation and VEGF-A evaluation

Blood was collected into BD Vacutainer® tubes (#366881, BD Biosciences, Plymouth, UK), allowed to clot for 1 h at 37 C°, centrifuged for 15 min at 1900xg at 4C° and then the serum aliquoted and stored at -80°C.

VEGF-A amount in serum samples was determined using Human VEGF DuoSet ELISA kit (DY293B, R&D Systems, Minneapolis, MN) according to the manufacturer’s instructions. All determinations were performed in duplicate.

### Statistical analysis

Statistical significance of the differences among experimental groups was assessed using unpaired or paired (cell cycle perturbation analysis) two-side Student’s *t* test. Significance was set at *P*<0.05.

Serum levels of VEGF-A were presented as medians and Interquartile Range (IQR) and were analyzed using non-parametric procedures. The Mann-Whitney U test was used to compare between-group differences, while the Wilcoxon matched-pairs signed-rank test was used to evaluate before-after differences. For data analysis, the value of 0.01 ng/mL was assigned to samples with undetectable VEGF-A level.

Receiver Operating Characteristics (ROC) analysis was performed for assessing the ability of VEGF-A serum levels to discriminate between patients who achieved CR or PR and patients who had SD or PD as their best response.

Statistical analyses were conducted using STATA11 (Stata Corp. LP, College Station, TX).

## Results

### Melanoma cells with acquired resistance to dabrafenib display changes in miRNA expression pattern

In order to identify miRNAs potentially involved in dabrafenib resistance and/or in the more aggressive phenotype that we had previously observed in melanoma cells resistant to this drug [27, 28], A375 and A375R cells were subjected to miRNA expression profiling and miRNAs differentially expressed between the two cell lines were identified by SAM analysis.

**Fig. 1:**
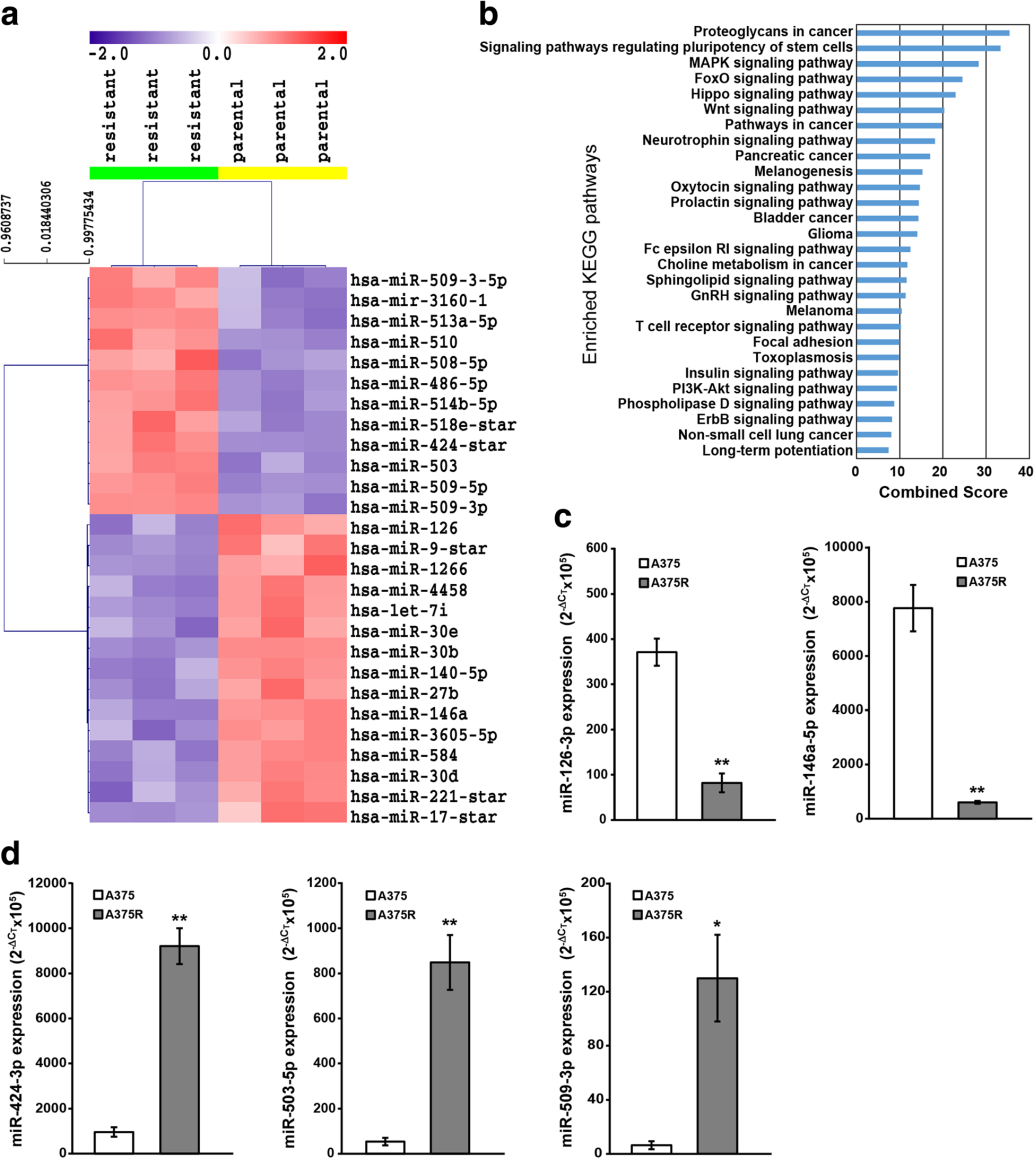
miRNAs differentially expressed between A375R and A375 cells and KEGG pathway analysis of putative targets. **a** Heatmap showing miRNA up-regulated (red = expression above the mean) and down-regulated (blue = expression below the mean) in dabrafenib-resistant A375R cells as compared with dabrafenib-sensitive A375 cells (SAM analysis; FC ≥ 2, FDR = 0%). miRNAs are indicated according to annotation provided by Affymetrix. **b** Putative target genes of differentially expressed miRNAs were obtained from TargetScan and used for KEGG pathway enrichment analysis. Only pathways with an adjusted *P* value <0.01 were considered and listed according to a decreasing value of the combined score. **c, d** Expression of miRNAs down-regulated (c) or up-regulated (d) in A375R cells according to microarray results was validated using specific TaqMan® MicroRNA Assays. The data were normalized to the level of RNU44 in each sample and expressed as 2^-ΔCt^x10^5^ values. Each value represents the arithmetic mean of at least three independent experiments performed with duplicate samples. Bars, standard error of the mean (SEM). ^**^P<0.01 and ^*^P<0.05, A375R versus (*vs*) A375.

Twenty-seven miRNAs were found to have 2-fold or greater differences in levels in A375R cell line as compared with its drug-sensitive counterpart. Data are presented in Fig. 1a as a heatmap showing miRNAs up-regulated (n =15) and down-regulated (n = 12) in A375R with respect to A375 cells.

We then analyzed the annotations of target mRNAs of the differentially expressed miRNAs for the enrichment of KEGG pathways. Several pathways previously involved in resistance to BRAFi and/or regulation of cell growth and invasiveness were enriched, including MAP kinase-, PI3K/AKT-, ErbB- [6, 7], Hippo- [37], WNT- [38], focal adhesion- [39], insulin- [40] signaling pathways (Fig. 1b).

To assess the robustness of the microarray results, we chose five differentially expressed miRNAs, namely miR-126-3p (fold change, FC=0.14), miR-146a-5p (FC=0.03), miR-424-3p (FC=5.7), miR-503-5p (FC=4.7), and miR-509-3p (FC=36), and analyzed their expression in A375 and A375R cells by qRT-PCR. The five miRNAs were selected based on their known involvement in the onset and/or progression of melanoma [12, 15, 26, 41, 42], and tumor chemoresistance [43–45]. qRT-PCR results confirmed the miRNAs to be either down-regulated (miR-126-3p, miR-146a-5p) (Fig. 1c), or up-regulated (miR-424-3p, miR-503-5p, miR-509-3p) (Fig. 1d), in A375R with respect to A375 cells.

**Fig. 2:**
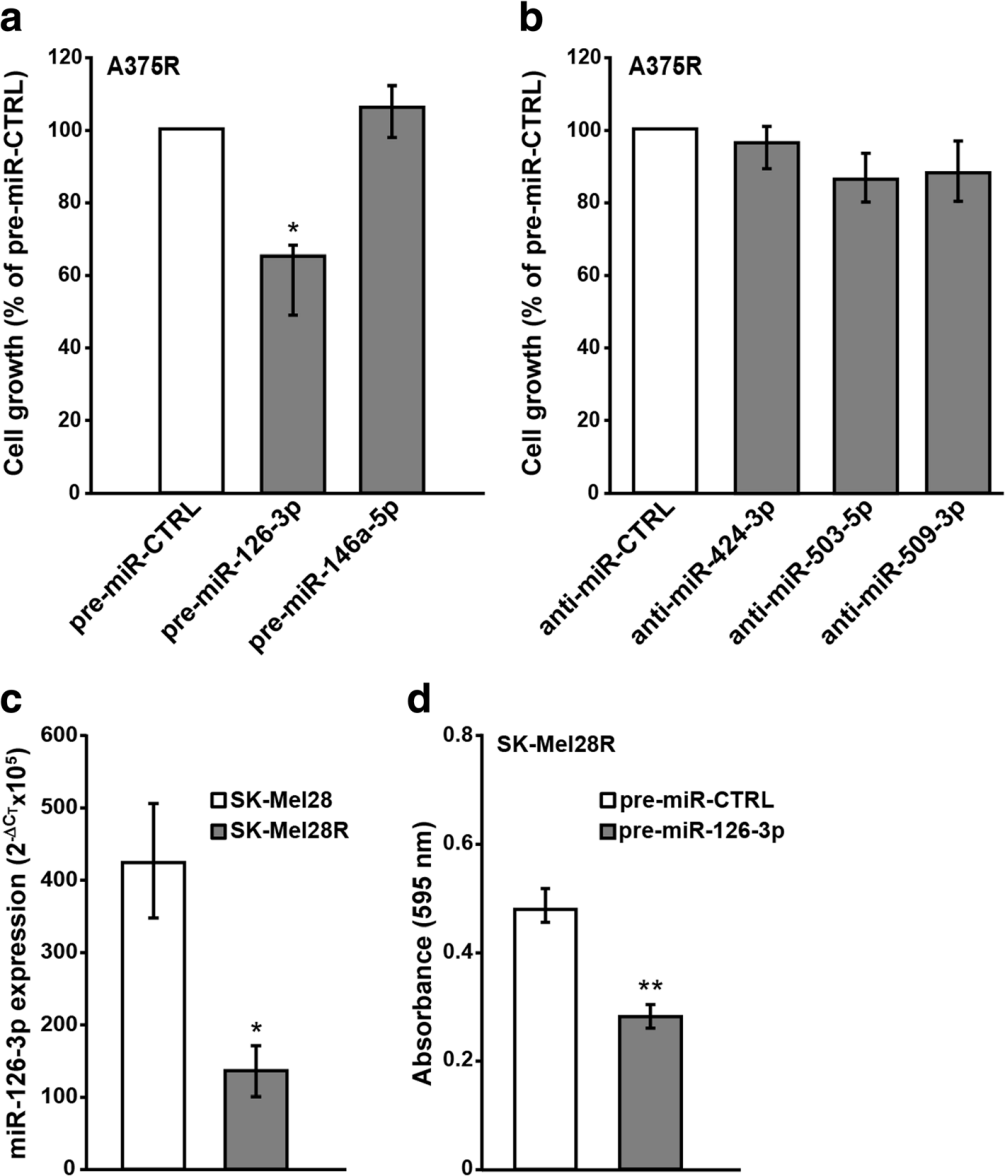
miR-126-3p replacement inhibits proliferation of dabrafenib-resistant cells. **a, b** A375R cells were transiently transfected with 50 nM of the indicated pre-miRs (a) or anti-miRs (b) or the appropriate controls, cultured for six days and then assayed for proliferation by the MTT assay. Data are expressed in terms of percentage of cell growth as compared to pre-miR-CTRL- or anti-miR-CTRL-transfected cells. Each value represents the arithmetic mean of three independent experiments performed with triplicate samples. Bars, SEM. ^*^P<0.05 A375R/pre-miR-126-3p *vs* A375R/pre-miR-CTRL. **c** qRT-PCR analysis of miR-126-3p expression in SK-Mel28 and SK-Mel28R cells. The data were normalized to the level of RNU44 in each sample and expressed as 2^-ΔCt^x10^5^ values. Each value represents the arithmetic mean of four independent experiments performed with duplicate samples. Bars, SEM. ^*^P<0.05, SK-Mel28R *vs* SK-Mel28. **d** SK-Mel28R cells were transiently transfected with 50 nM pre-miR-126-3p or pre-miR-CTRL, cultured for six days and then assayed for proliferation by the MTT assay. Data are expressed in terms of absorbance at 595 nM. Each value represents the arithmetic mean of four independent experiments performed with triplicate samples. Bars, SEM. ^**^P<0.01, SK-Mel28R/pre-miR-126-3p *vs* SK-Mel28R/pre-miR-CTRL.

We next investigated whether replacement of the down-regulated miRNAs or inhibition of the up-regulated miRNAs could affect A375R cell proliferation. As illustrated in Fig. 2a and Fig. 2b, only transfection of pre-miR-126-3p significantly affected A375R cell proliferation. Based on those results, we selected miR-126-3p for further studies. In a first set of experiments we evaluated whether miR-126-3p was down-regulated also in a different dabrafenib-resistant melanoma cell line (i.e. SK-Mel28R) and whether its replacement was able to impair cell proliferation. Indeed, the expression of miR-126-3p resulted markedly reduced in SK-Mel28R cells as compared with SK-Mel28 parental cells (Fig. 2c) and transfection of pre-miR-126-3p into SK-Mel28R cells significantly inhibited proliferation (Fig. 2d).

### miR-126-3p replacement affects cell cycle progression of dabrafenib-resistant cells and reduces the levels of phosphorylated ERK1/2 and AKT

**Fig. 3:**
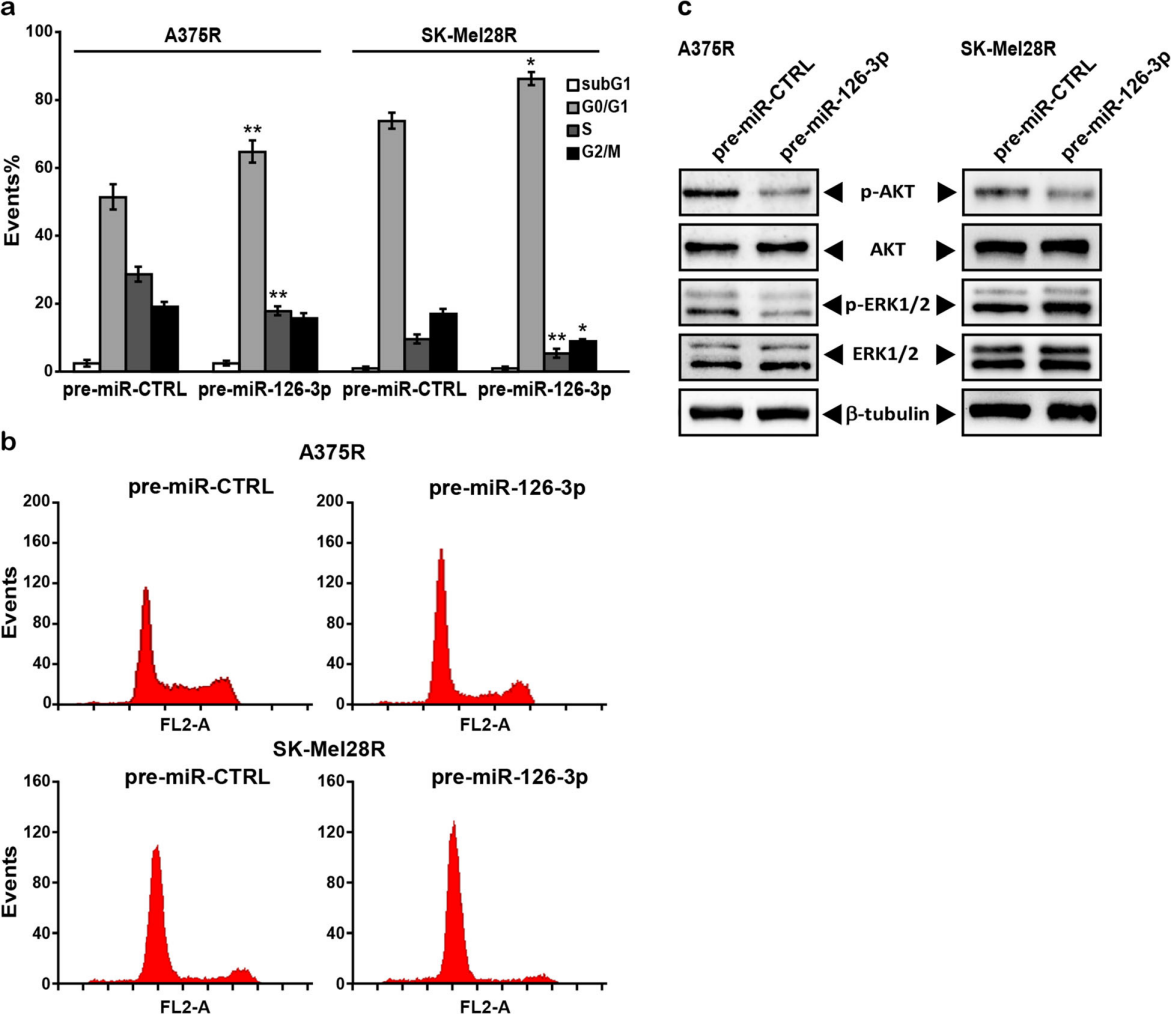
miR-126-3p replacement induces G0/G1 arrest in dabrafenib-resistant cells and decreases the levels of phosphorylated ERK1/2 and/or AKT. **a** Melanoma cells were transiently transfected with 50 nM pre-miR-126-3p or pre-miR-CTRL and 72 h later removed from continuous culture and analyzed for apoptosis induction and cell cycle perturbations. Each value represents the arithmetic mean of four (A375R) or three (SK-Mel28R) independent experiments. Bars, SEM. ^**^P<0.01 and ^*^P<0.05, pre-miR-126-3p *vs* matched pre-miR-CTRL. **b** Flow cytometry profiles relative to a representative experiment. **c** Melanoma cells were transfected as described in (a) and 48 h later cell lysates were analyzed by immunoblotting using antibodies against pospho-ERK1/2 (Thr202/Tyr204) (p-ERK1/2), ERK1/2, phospho-AKT (Ser473) (p-AKT), AKT or β-tubulin. The results are representative of two independent experiments giving comparable results.

We next investigated whether the growth inhibitory effects of miR-126-3p replacement observed in the dabrafenib-resistant cell lines were due to the induction of apoptosis and/or cell cycle perturbations and whether activation of the MAPK and PI3K/AKT pathways was impaired by miR-126-3p re-expression. As illustrated in Fig. 3a-b, no apoptosis was detected in A375R and SK-Mel28R cells transfected with pre-miR-126-3p (hereafter also referred to as pre-miR-126-3p/A375R and pre-miR-126-3p/SK-Mel28R cells). On the other hand, in both cell lines miR-126-3p replacement was associated with a significant increase in the percentage of cells in the G0/G1 phase of the cell cycle, and a concomitant decrease in the percentages of cells in the S phase. In pre-miR-126-3p/SK-Mel28R cells, a reduction of the G2/M population was also observed. Moreover, Western blot analysis evidenced a decrease in the levels of phosphorylated AKT in both pre-miR-126-3p-transfected cell lines, and a reduction of phosphorylated ERK1/2 in pre-miR-126-3p/A375R cells (Fig. 3c).

### miR-126-3p replacement affects response of resistant cells to dabrafenib

**Fig. 4:**
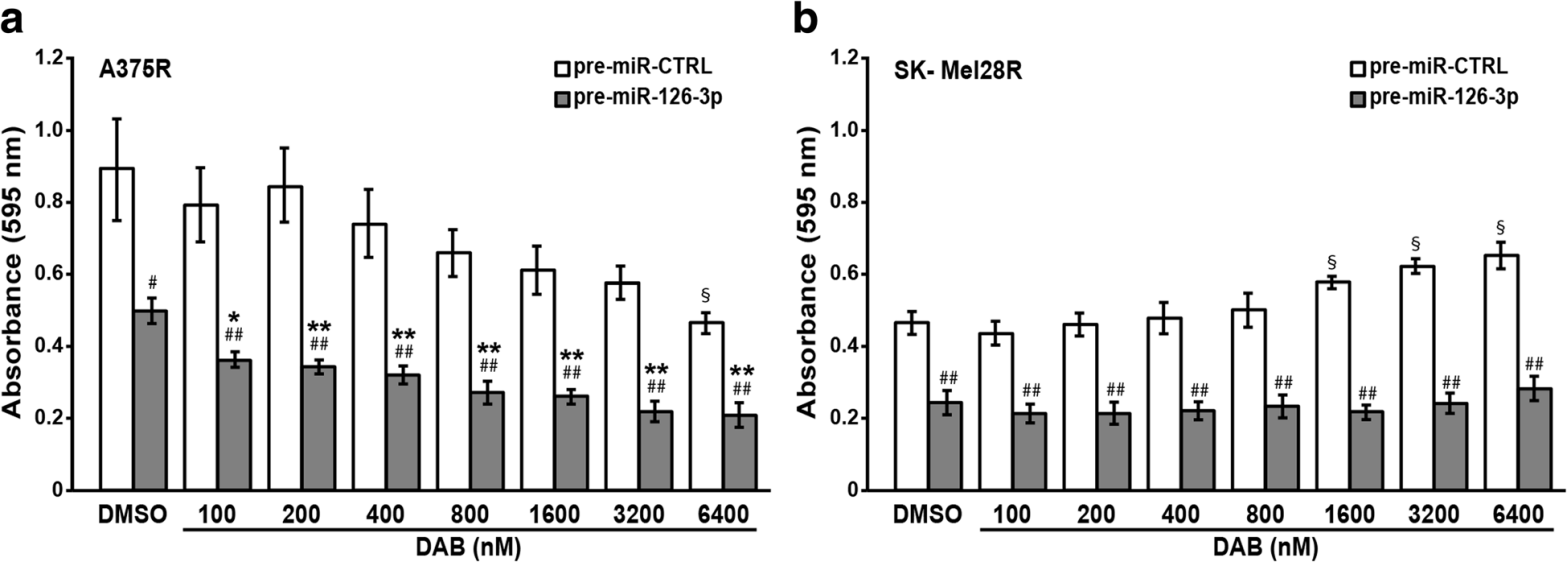
miR-126-3p replacement increases response of drug-resistant cells to dabrafenib. **a**, **b** Melanoma cells were transiently transfected with 50 nM pre-miR-126-3p or pre-miR-CTRL and 24 h later incubated with graded concentrations of dabrafenib (DAB) or with DMSO alone. After five days of culture, proliferation was assessed by the MTT assay. Data are expressed in terms of absorbance at 595 nM. Each value represents the arithmetic mean of four (A375R) or five (SK-Mel28R) independent experiments performed with triplicate samples. Bars, SEM. ^##^*P*<0.01 and ^#^*P*<0.05, pre-miR-126-3p *vs* matched pre-miR-CTRL; ^**^P<0.01 and ^*^P<0.05 pre-miR-126-3p/DAB *vs* pre-miR-126-3p/DMSO; ^§^*P*<0.05 pre-miR-CTRL/DAB *vs* pre-miR-CTRL/DMSO.

Having demonstrated that miR-126-3p was down-regulated in dabrafenib-resistant cells and that its replacement impaired cell proliferation, we investigated whether re-expression of miR-126-3p could also attenuate cell resistance to dabrafenib. pre-miR-CTRL/A375R cells showed a statistically significant inhibition of proliferation only at a drug concentration of 6400 nM, whereas pre-miR-126-3p/A375R cells displayed a further reduction of proliferation at all drug concentrations tested (Fig. 4a). In agreement with our previous findings [27], proliferation of pre-miR-CTRL/SK-Mel28R cells was not affected by dabrafenib concentrations up to 800 nM and even stimulated at drug concentrations ranging between 1600 and 6400 nM. In pre-miR-126-3p/SK-Mel28R cells, the growth promoting effect of dabrafenib was no more evident and proliferation remained comparable to that of DMSO-treated cells at all drug concentrations (Fig. 4b).

### Dabrafenib induces up-regulation of miR-126-3p in A375 and SK-Mel28 cells but not in their drug-resistant counterparts

**Fig. 5:**
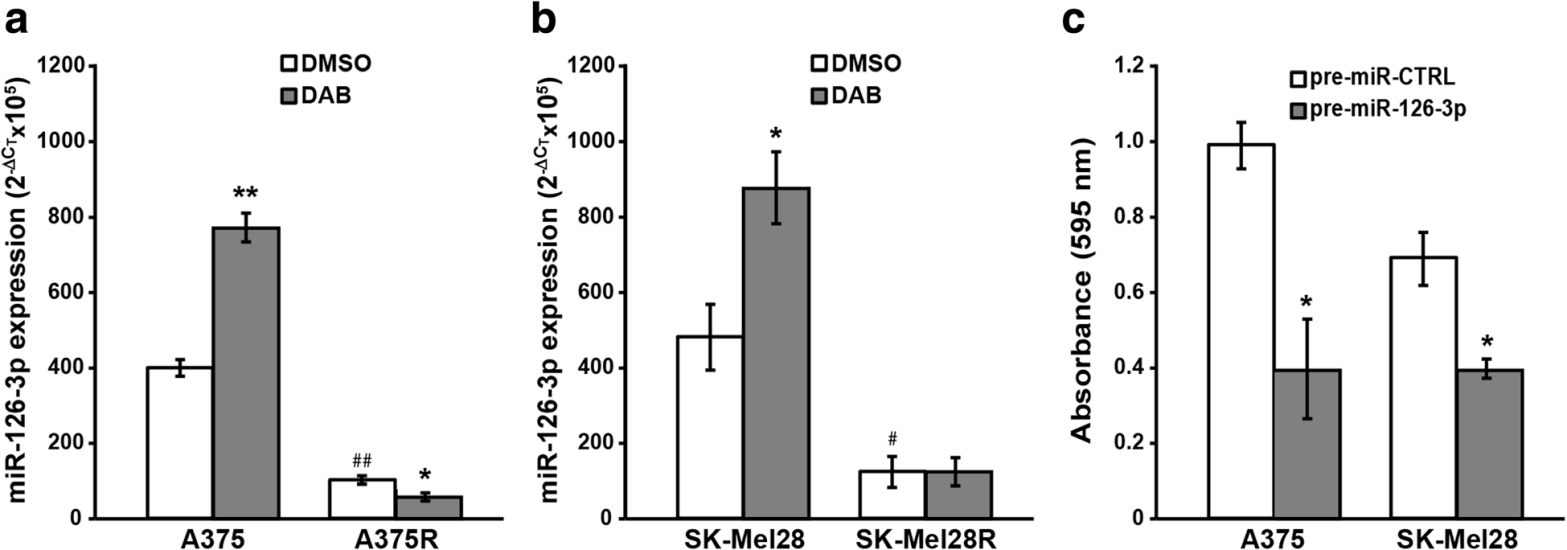
miR-126-3p is up-regulated by dabrafenib only in drug-sensitive cells and inhibits their proliferation. **a, b** Melanoma cells were incubated with 100 nM dabrafenib (DAB) or with DMSO alone and after 48 h of culture miR-126-3p expression was evaluated by qRT-PCR. 2^-ΔCt^x10^5^ values calculated relative to RNU44 as the internal reference are shown. Each value represents the arithmetic mean of three independent experiments performed with duplicate samples. Bars, SEM. ^**^P<0.01 and ^*^P<0.05, DAB *vs* matched DMSO; ^##^*P*<0.01 and ^#^*P*<0.05, resistant cells/DMSO *vs* parental cells/DMSO. **c** A375 and SK-Mel28 were transiently transfected with 50 nM pre-miR-126-3p or pre-miR-CTRL, cultured for six days and then assayed for proliferation by the MTT assay. Data are expressed in terms of absorbance at 595 nM. Each value represents the arithmetic mean of three (A375) or four (SK-Mel28) independent experiments performed with triplicate samples. Bars, SEM. ^*^P<0.05, pre-miR-126-3p *vs* pre-miR-CTRL.

Based on the findings that miR-126-3p was down-regulated in dabrafenib-resistant cells and when re-expressed inhibited cell proliferation and reduced drug resistance, we hypothesized that growth inhibition induced by dabrafenib in drug-sensitive cells could result, at least in part, from drug-induced up-regulation of miR-126-3p and that cells with acquired resistance to dabrafenib failed to up-regulate this miRNA in response to drug treatment. Consistent with this hypothesis, we found that a short-term exposure to dabrafenib markedly up-regulated miR-126-3p expression in A375 and SK-Mel28 cell lines, whereas the levels of the miRNA were moderately reduced in A375R cells (Fig. 5a) and not affected in SK-Mel28R cells (Fig. 5b). Moreover, transfection of pre-miR-126-3p was able to significantly inhibit proliferation of A375 and SK-Mel28 cells (Fig. 5c).

### Enforced expression of miR-126-3p in dabrafenib-sensitive cells delays the onset of secondary resistance to the drug

To further address the role of miR-126-3p down-regulation in acquired resistance to dabrafenib, we performed a long-term proliferation assay to evaluate whether enforced miR-126-3p expression during chronic exposure of melanoma cells to dabrafenib was able to delay the emergence of resistance. To this end, SK-Mel28 cells were co-treated with pre-miR-126-3p and dabrafenib every eight days over a period of 96 days and monitored for proliferation.

**Fig 6:**
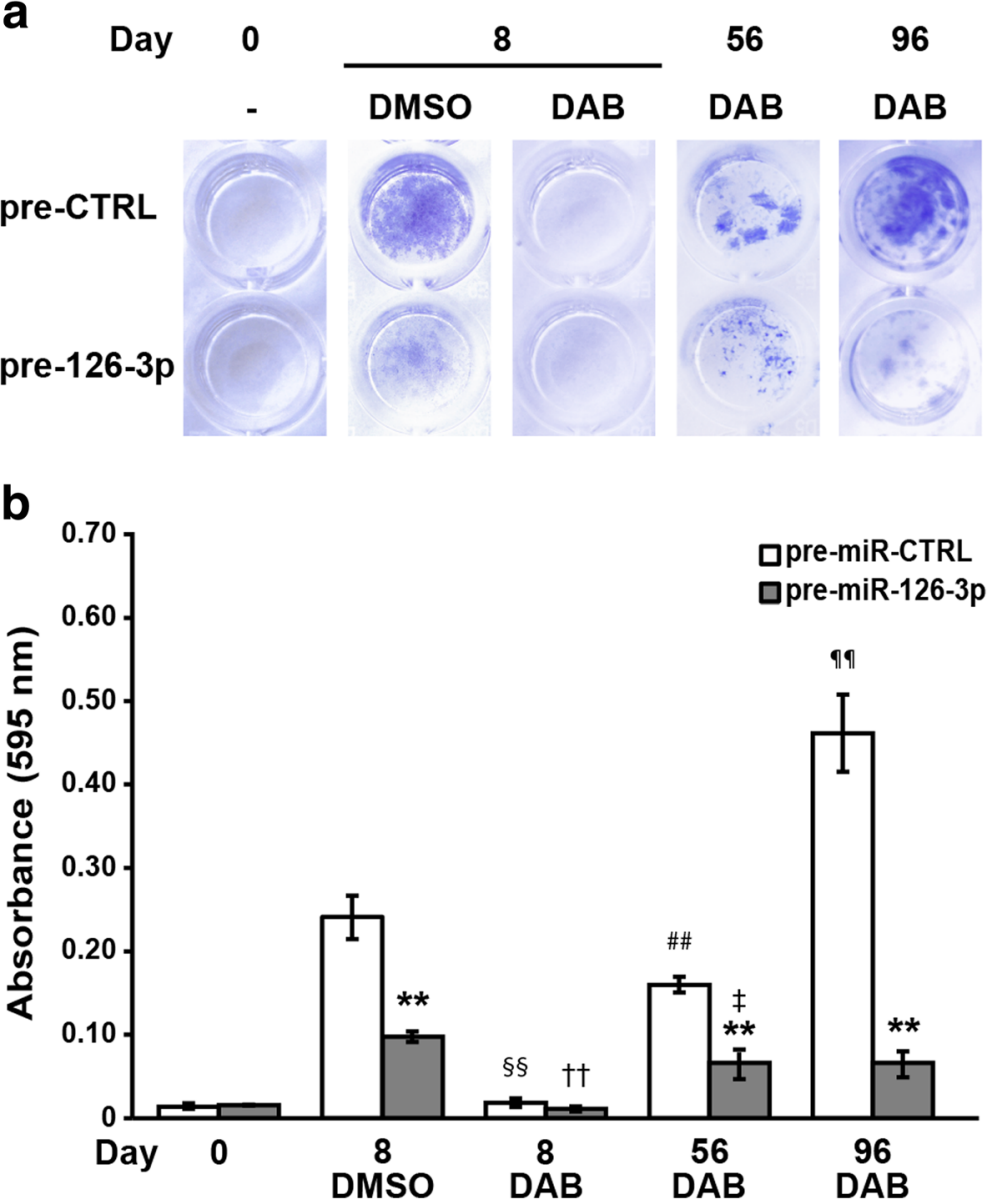
Enforced expression of miR-126-3p delays the development of resistance to dabrafenib**. a** SK-Mel28 cells were seeded into 96-well plates and every eight days transfected with 50 nM pre-miR-126-3p (pre-126-3p) or pre-miR-CTRL (pre-CTRL) and treated with 100 nM dabrafenib (DAB) or DMSO. Cell cultures were photographed and processed for quantitative analysis of proliferation on day 0 (i.e after the first transfection), 8, 56 and 96. Images from a representative experiment are shown. **b** Quantitative analysis of proliferation of cell cultures described in (a). Crystal violet was solubilized and absorbance was read at 595 nm. Each value represents the arithmetic mean of two independent experiments performed with triplicate cultures. Bars, SEM. ^**^P<0.01, pre-miR-126-3p *vs* matched pre-miR-CTRL; ^§§^*P*<0.01, pre-miR-CTRL/DAB/Day 8 *vs* pre-miR-CTRL/DMSO/Day 8; ^††^*P*<0.01, pre-miR-126-3p/DAB/Day 8 *vs* pre-miR-126-3p/DMSO/Day 8; ^##^*P*<0.01, pre-miR-CTRL/DAB/Day 56 *vs* pre-miR-CTRL/DAB/Day 8; ^¶¶^*P*<0.01, pre-miR-CTRL/DAB/Day 96 *vs* pre-miR-CTRL/DAB/Day 56; ^‡^pre-miR-126-3p/DAB/Day 56 *vs* pre-miR-126-3p/DAB/Day8.

The results illustrated in Fig. 6a-b confirmed that enforced expression of miR-126-3p alone was able to impair SK-Mel28 cell proliferation (day 8, DMSO group). A marked and comparable inhibition of cell growth was observed on day 8 in pre-miR-CTRL/SK-Mel28 and pre-miR-126-3p/SK-Mel28 cells exposed to dabrafenib. However, while drug-treated pre-miR-CTRL/SK-Mel28 cells clearly started to regrowth after the initial inhibition of proliferation, drug-treated pre-126-3p/SK-Mel28 cells displayed only a marginal recovery of proliferation even on day 96 of culture, consistent with a delay in the development of secondary resistance (Fig. 6a-b).

### Replacement of miR-126-3p impairs invasiveness of dabrafenib-resistant cells

**Fig. 7:**
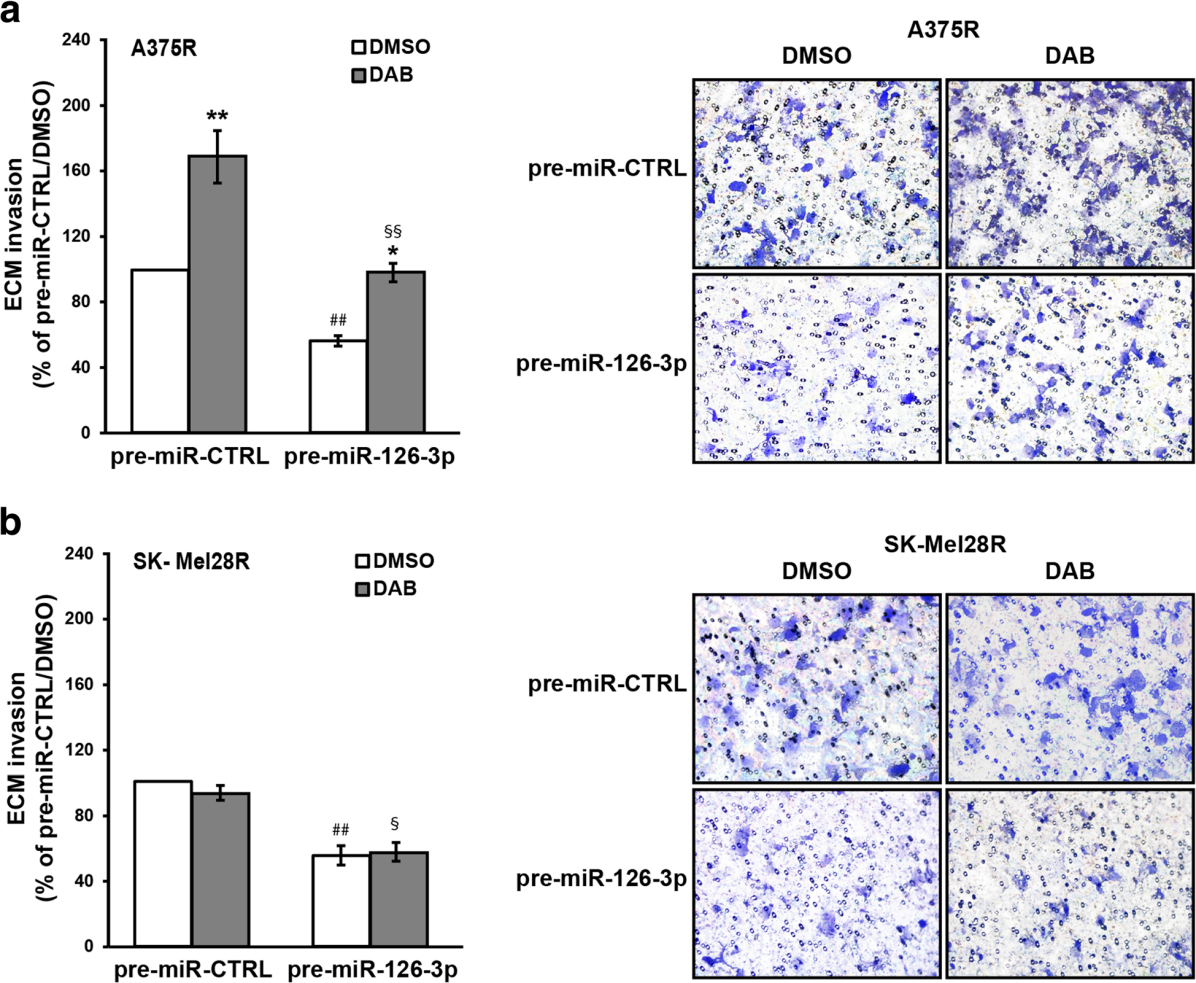
miR-126-3p replacement impairs invasiveness of dabrafenib-resistant cells. **a**, **b** Melanoma cells were transiently transfected with 50 nM pre-miR-126-3p or pre-miR-CTRL and 24 h later incubated with 100 nM dabrafenib (DAB) or DMSO alone. After additional 48 h of culture, the cells were assayed for ECM invasion. Left panels, percentage of invaded cells with respect to DMSO-treated/pre-miR-CTRL-transfected cells. Each value represents the arithmetic mean of four (A375R) or three (SK-Mel28R) independent experiments performed with triplicate samples. Bars, SEM. ^##^*P*<0.01, pre-miR-126-3p/DMSO *vs* pre-miR-CTRL/DMSO; ^§§^*P*<0.01 and ^§^*P*<0.05, pre-miR-126-3p/DAB *vs* pre-miR-CTRL/DAB; ^**^P<0.01 and ^*^P<0.05, DAB *vs* matched DMSO. Right panels, representative images of polycarbonate filters with invaded melanoma cells.

We previously demonstrated that A375R and SK-Mel28R cell lines are more invasive than their matched parental cell lines [27, 28]. Moreover, while exposure to dabrafenib (100 nM) significantly inhibited invasiveness of A375 and SK-Mel28 cells, the drug did not affect the invasive capacity of SK-Mel28R cells and further stimulated that of A375R cells [27, 28]. On the other hand, previous studies by Felli et al [26] demonstrated that enforced expression of miR-126-3p&5p caused a significant reduction of invasiveness in two different melanoma cell lines. We, therefore, evaluated whether replacement of miR-126-3p could also affect the invasive capacity of dabrafenib-resistant cells under basal condition or in response to dabrafenib. As shown in Fig. 7a-b, transfection of pre-miR-126-3p alone was able to reduce the invasive capacity of A375R and SK-Mel28R cells of about 40%. Exposure to dabrafenib promoted extracellular matrix (ECM) invasion in both pre-miR-CTRL/A375R and pre-miR-126-3p/A375R cells. However, invasiveness of drug-treated pre-miR-126-3p/A375R cells was significantly lower than that of drug-treated pre-miR-CTRL/A375R cells and comparable to that of cells transfected with pre-miR-CTRL and treated with DMSO (Fig. 7a). As expected from our previous studies [27], exposure to dabrafenib did not modify the invasive capacity of pre-miR-CTRL/SK-Mel28R cells. Conversely, both DMSO- and dabrafenib-treated pre-miR-126-3p/SK-Mel28R cells displayed inhibition of ECM invasion (Fig. 7b). Therefore, restoration of miR-126-3p was able to impair invasiveness also in dabrafenib-resistant cells.

### ADAM9 is a target of miR-126-3p in dabrafenib-resistant cells and its silencing impairs proliferation and invasiveness and increases dabrafenib sensitivity

To identify targets regulated by miR-126-3p that could affect proliferation, invasiveness and response to dabrafenib of the drug-resistant cells, we focused our attention on *ADAM9* (a disintegrin and a metalloproteinase domain 9), *MMP7* (matrix metallopeptidase 7) and *PIK3R2* (phosphoinositide-3-kinase regulatory subunit 2), three miR-126-3p target genes already validated in melanoma cells [26] and shown to be involved in melanoma invasiveness (*ADAM9* and *MMP7*) [26] and acquired resistance to BRAFi (*PIK3R2*) [6]. We also selected *CXCR4* (C-X-C motif chemokine receptor 4), since this gene was shown to be a direct target of miR-126-3p in colon and thyroid cancer cells [42, 46] and the CXCR4/CXCL12 (C-X-C motif chemokine ligand 12) axis is known to promote melanoma cell proliferation and invasiveness [47].

**Fig. 8:**
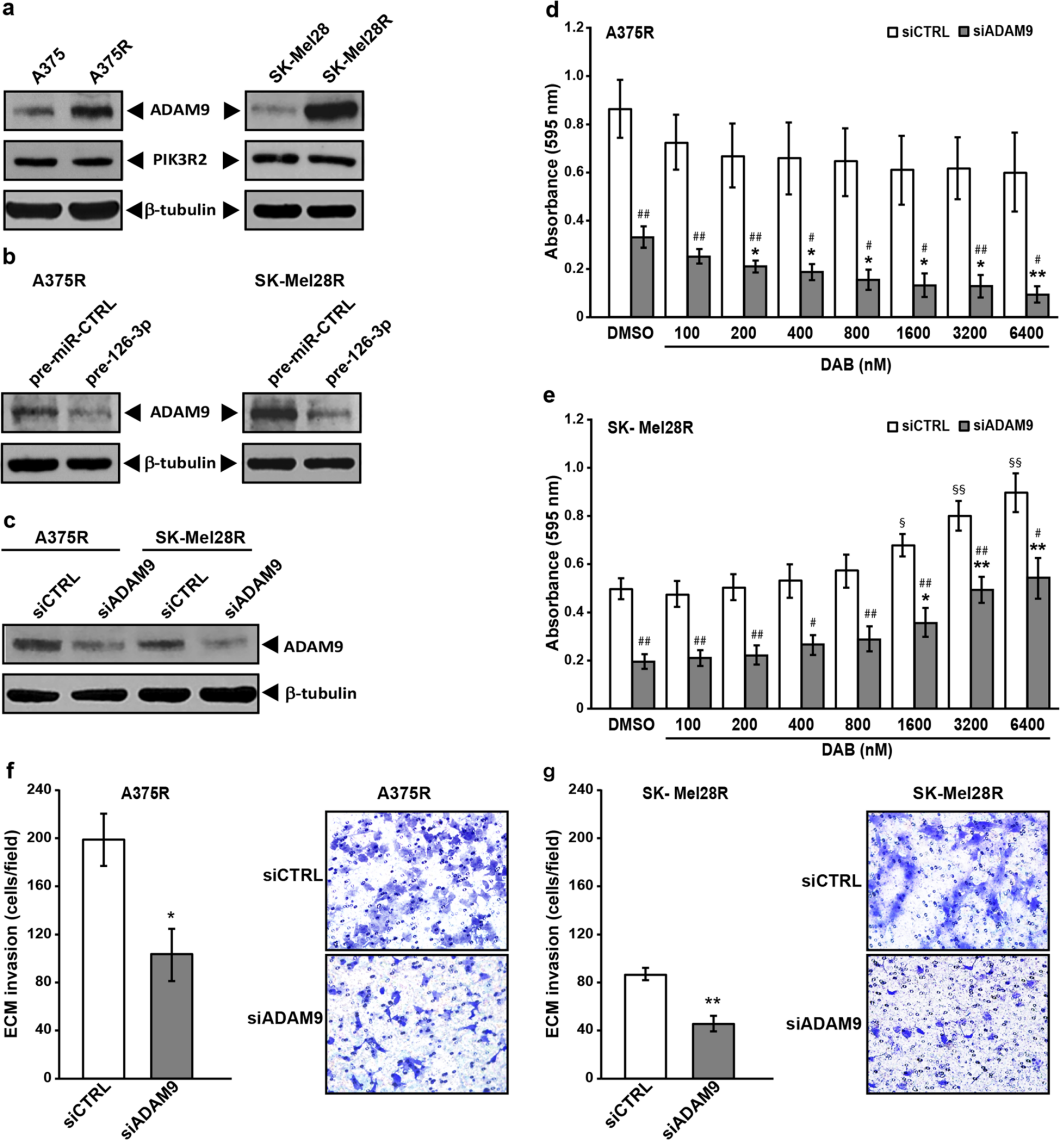
*ADAM9* silencing impairs proliferation and invasiveness and increases drug sensitivity of dabrafenib-resistant cells. **a** Melanoma cell lysates were analyzed by immunoblotting using antibodies against ADAM9 and PIK3R2, or against β-tubulin as a loading control. The results are representative of three independent experiments. **b** Melanoma cells were transfected with 50 nM pre-miR-126-3p (pre-126-3p) or pre-miR-CTRL and assayed for ADAM9 and PIK3R2 expression 72 h after transfection. **c** Melanoma cells were transfected with 10 nM (SK-Mel28R) or 50 nM (A375R) siADAM9 or siCTRL and assayed for ADAM9 and PIK3R2 expression 72 after transfection. **d, e** Melanoma cells were transfected as in (c) and 24 h later incubated with graded concentrations of dabrafenib (DAB) or with DMSO alone. After five days of culture, proliferation was assessed by the MTT assay. Data are expressed in terms of absorbance at 595 nM. Each value represents the arithmetic mean of five independent experiments performed with triplicate samples. Bars, SEM. ^##^*P*<0.01 and ^#^*P*<0.05, siADAM9 *vs* matched siCTRL; ^**^P<0.01 and ^*^P<0.05, siADAM9/DAB *vs* siADAM9/DMSO; ^§§^*P*<0.01 and ^§^*P*<0.05, siCTRL/DAB *vs* siCTRL/DMSO. **f, g** Melanoma cells were transfected as in (c) and 72 h after transfection tested for ECM invasion. Left panels, number of invaded cells per microscopic field. Each value represents the arithmetic mean of four (A375R) or five (SK-Mel28R) independent experiments performed with triplicate samples. Bars, SEM. ^**^P<0.01 and ^*^P<0.05, siADAM9 *vs* siCTRL. Right panels, representative images of polycarbonate filters with invaded melanoma cells.

We initially analyzed the expression level of the proteins encoded by the four genes in A375, A375R, SK-Mel28 and SK-Mel28R cells. With respect to the corresponding parental cells, ADAM9 was up-regulated in the dabrafenib-resistant cells, whereas PIK3R2 was expressed at comparable levels (Fig. 8a), and MMP7 was down-regulated (Additional file 1: Figure S1). Down-regulation of CXCR4 protein levels was observed in SK-Mel28R cells but not in A375R (Additional file 1: Figure S1).

Based on these results, that supported a role of ADAM9 but not of the other proteins in acquired resistance to dabrafenib, we selected ADAM9 for further experiments and evaluated whether this protein was down-regulated in A375R and SK-Mel28R cells after transfection with pre-miR-126-3p. As illustrated in Fig. 8b, miR-126-3p replacement markedly inhibited ADAM9 expression in both cell lines. ADAM9 expression was also reduced in A375 and SK-Mel28 parental cells treated with 100 nM dabrafenib (Additional file 2: Figure S2), in agreement with the finding that the drug up-regulates miR-126-3p in those cells.

We next investigated whether direct targeting of *ADAM9* through specific siRNAs could reduce proliferation and/or increase dabrafenib sensitivity of A375R and SK-Mel28R cells.

The two cell lines were, therefore, transfected with siADAM9 or a siCTRL, incubated with increasing concentrations of dabrafenib 24 h after transfection, and assayed for proliferation five days later using the MTT assay. To confirm inhibition of ADAM9 expression, the protein levels were determined by Western blotting.

In both cell lines, siADAM9 reduced the level of the target protein (Fig. 8c) and significantly impaired proliferation (Fig. 8d-e, DMSO groups). Dabrafenib treatment did not substantially affect proliferation of siCTRL/A375R cells, whereas it further reduced that of siADAM9/A375R cells, starting from the concentration of 200 nM (Fig. 8d). siCTRL/SK-Mel28R and siADAM9/SK-Mel28R cells displayed a similar behavior in response to dabrafenib, as their proliferation was unaffected by drug concentrations up to 800 nM and stimulated at higher concentrations. Nevertheless, proliferation of siADAM9/SK-Mel28R cells was always significantly lower than that of siCTRL/SK-Mel28R cells at each dabrafenib concentration, and did not exceeded that of DMSO-treated siCTRL-transfected cells at drug concentrations ranging between 800 nM and 1600 nM (Fig. 8e).

We next evaluated whether ADAM9 could be involved in the regulation of the invasive capacity of dabrafenib-resistant cells. As illustrated in Fig. 8f-g, *ADAM9* knock-down caused a significant inhibition of the invasive capacity of both A375R and SK-Mel28R cell lines.

### *ADAM9* silencing delay the establishment of dabrafenib resistance

**Fig. 9:**
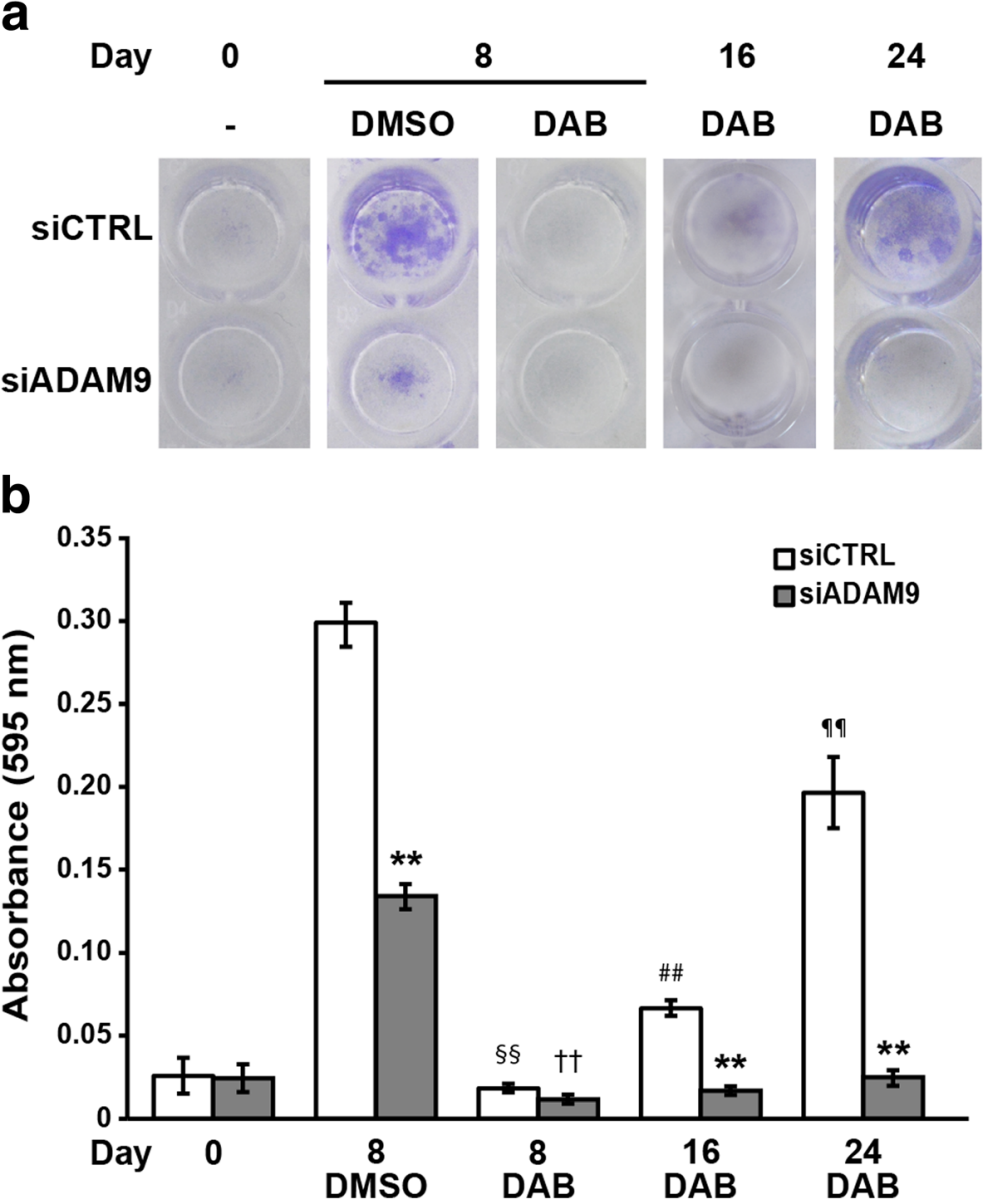
*ADAM9* silencing delays the development of resistance to dabrafenib. A375 cells were seeded into 96-well plates and every eight days transfected with 50 nM siADAM9 or siCTRL and treated with 100 nM dabrafenib (DAB) or DMSO. Cell cultures were photographed and processed for quantitative analysis of proliferation on day 0 (i.e after the first transfection), 8, 16 and 24. Images from a representative experiment are shown. **b** Quantitative analysis of proliferation of cell cultures described in (a). Crystal violet was solubilized and absorbance was read at 595 nm. Each value represents the arithmetic mean of three independent experiments performed with triplicate cultures. Bars, SEM. ^**^P<0.01, siADAM9 *vs* matched siCTRL; ^§§^*P*<0.01, siCTRL/DAB/Day 8 *vs* siCTRL/DMSO/Day 8; ^††^*P*<0.01, siADAM9/DAB/Day 8 *vs* siADAM9/DMSO/Day8; ^##^*P*<0.01, siCTRL/DAB/Day 16 *vs* siCTRL/DAB/Day 8; ^⁋⁋^*P*<0.01, siCTRL/DAB/Day 24 *vs* siCTRL/DAB/Day 16.

Based on the findings that ADAM9 was a target of miR-126-3p in A375R and SK-Mel28R cells and that its silencing attenuated resistance to dabrafenib, we investigated whether negative modulation of ADAM9 in A375 cells could antagonize the development of secondary resistance to this drug. Using a long-term proliferation assay, we confirmed that inhibition of ADAM9 expression impaired proliferation of A375 cells, as evaluated in DMSO-treated cells 8 days after transfection (Fig. 9a-b). A strong inhibition of proliferation was observed on day 8 in both siCTRL/A375 and siADAM9/A375 cells exposed to dabrafenib. However, while proliferation of drug-treated siCTRL/A375 cells gradually resumed from day 16 to day 24, that of drug-treated siADAM9/A375 cells remained markedly inhibited, consistent with a delay in the development of secondary resistance (Fig. 9a-b). Comparable results were obtained in a different set of experiments performed with SK-Mel28 cells (Additional file 3: Figure S3).

### *VEGFA* is a target of miR-126-3p in dabrafenib-resistant melanoma cells and its silencing impairs cell proliferation and invasiveness

Autocrine loops sustained by the interaction of VEGF-A with its receptors promote melanoma cell proliferation and invasiveness [48–50]. VEGF-A has also been validated as a direct miR-126 target gene in several types of human cancer [36, 42, 51], and down-regulation of VEGF-A mRNA has been observed in melanoma cells over-expressing miR-126-3p&5p [26]. Moreover, we previously showed that dabrafenib markedly down-regulated VEGF-A expression in A375 and SK-Mel28 cells [27, 28]. We therefore investigated whether replacement of miR-126-3p in A375R and SK-Mel28R caused a down-regulation of VEGF-A expression and whether this molecular event was involved in the inhibitory effects exerted by miR-126-3p on cell proliferation and invasiveness.

**Fig. 10:**
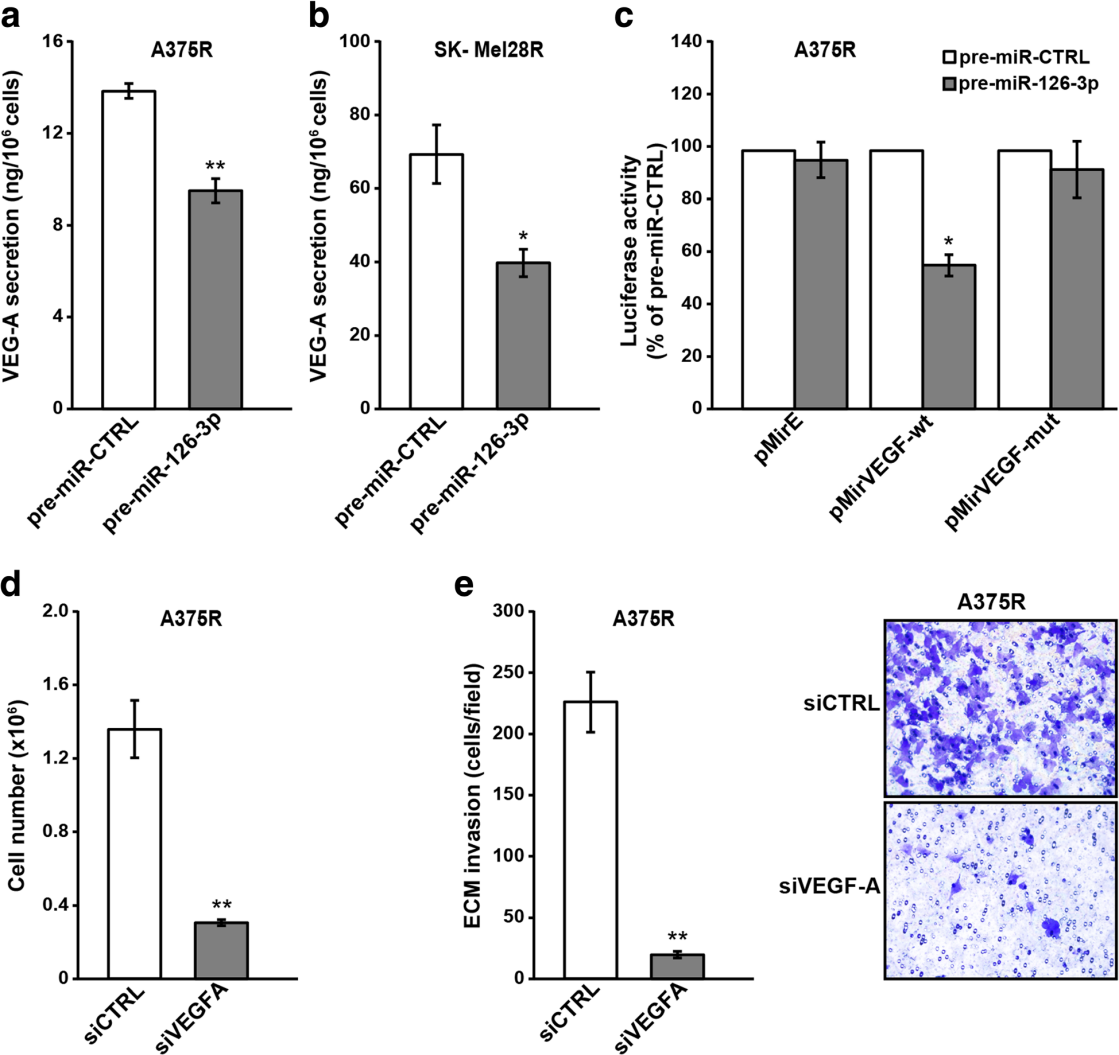
*VEGFA* is a miR-126-3p target and its silencing impairs proliferation and invasiveness of dabrafenib-resistant cells. **a, b** Melanoma cells were transiently transfected with 50 nM pre-miR-126-3p or pre-miR-CTRL and after 72 h of culture, cell supernatants were collected and the amount of VEGF-A quantified by ELISA. Each value represents the arithmetic mean of three (A375R) or four (SK-Mel28R) independent experiments performed with duplicate samples. Bars, SEM. ^**^P<0.01 and ^*^P<0.05, pre-miR-126-3p *vs* pre-miR-CTRL. **c** A375R cells were co-transfected with 100 ng of the indicated vectors and 10 ng of the pRL-null vector encoding *Renilla* luciferase along with 50 ng of pre-miR-126-3p or pre-miR-CTRL. Firefly luciferase levels were determined 48 h after transfection and normalized to those of *Renilla* luciferase. Data are expressed in terms of percentage of luciferase activity detected in pre-miR-126-3p-transfected cells with respect to that of pre-miR-CTRL-transfected cells. Each value represents the arithmetic mean of five independent experiments. Bars, SEM. ^*^P<0.05, pre-miR-126-3p *vs* pre-miR-CTRL. **d** A375R cells (7.5x10^4^) were plated into 6 cm-dishes and 18 h later transiently transfected with 50 nM siVEGFA or siCTRL. After 72 h of culture the cells were detached and counted. Each value represents the arithmetic mean of three independent experiments performed with duplicate samples. Bars, SEM. ^**^P<0.01, siVEGFA *vs* siCTRL. **e** A375R cells were plated and treated as in (d) and 72 h after transfection assayed for ECM invasion. Left panel, number of invaded cells per microscopic field. Each value represents the arithmetic mean of three independent experiments performed with triplicate samples. Bars, SEM. ^**^P<0.01, siVEGFA *vs* siCTRL. Right panel, representative images of polycarbonate filters with invaded melanoma cells.

A375R and SK-Mel28R cells were transfected with pre-miR-126-3p or pre-miR-CTRL and 72 h later the amount of VEGF-A in culture supernatants was determined by ELISA. As illustrated in Fig. 10a-b, re-expression of miR-126-3p produced a reduction of VEGF-A secretion in both A375R and SK-Mel28R cells.

To further confirm that VEGF-A is a direct target of miR-126-3p in melanoma cells, a 3′ UTR luciferase assay was carried out. A significant decrease in luciferase activity was observed in A375R cells co-transfected with pre-miR-126-3p and the pMirVEGF-wt vector. In contrast, pre-miR-126-3p did not influence the reporter activity of the pMirVEGF-mut construct, in which the miR-126-3p seed complementary sequence had been mutated (Fig. 10c).

Finally, A375R cells were transfected with siVEGFA or siCTRL and VEGF-A secretion, proliferation and ECM invasion were evaluated after 72 h of culture. VEGF-A secretion was almost abrogated (98±1% inhibition) in siVEGFA/A375R cells. Moreover, both cell proliferation and ECM invasion were strongly inhibited by *VEGFA* silencing (Fig. 10d-e).

### Evaluation of VEGF-A serum levels in melanoma patients subjected to therapy with BRAFi or BRAFi+MEKi

VEGF-A is a potent inducer of angiogenesis, a growth and metastasis promoting factor in melanoma, and also contributes to the suppression of antitumor responses through inhibition of dendritic cell maturation and T-cell responses [52]. Elevated VEGF-A serum levels have been shown to be a negative prognostic factor in melanoma patients [53]. Moreover, in melanoma patients treated with high-dose interleukin-2 [54] or ipilimumab [55], high VEGF-A serum levels were found to be associated with poor clinical response and decreased overall survival. Based on those previous findings and our in vitro results, we carried out a pilot study in patients receiving BRAFi or BRAFi+MEKi therapy to determine whether VEGF-A serum levels detected before the start of treatment and/or changes in that cytokine levels during therapy were associated with clinical response.

The study was conducted on a total of 26 patients from whom serum samples collected before the start of the therapy (T0), after two months of treatment (T2) and at disease progression (TP) were available. Among the 26 patients, 11 were subjected to dabrafenib or vemurafenib monotherapy, 13 were treated with dabrafenib plus trametinib, 1 with vemurafenib plus cobimetinib and 1 with dabrafenib alone for 5 months and then with dabrafenib plus trametinib (Table 1). Six patients had PD as best response, 2 had SD, 17 had PR and 1 had CR (Table 1). Patients experiencing PR or CR constituted the group of “responders”, whereas patients with PD or SD as best response were included in the group of “non-responders” and only T0 and T2 serum samples were considered for the analysis.

**Table 1:** Demographic and clinical characteristics of melanoma patients from whom serum was collected

Case	Sex	Age (years)	Stage^a^	LDH^b^	Previous therapy	Targeted therapy^c^	BR^d^	TTF^e^ (days)
1	F	48	M1c	H	Fotemustine	DAB	PR	182
2	M	38	M1c	H	None	DAB	PR	144
3	F	64	M1b	N	None	DAB+TRAM	PR	234
4	M	71	M1c	N	None	DAB (5)➔DAB+TRAM	PR	1275
5	M	43	M1c	H	None	DAB	SD	148
6	M	47	M1c	H	Fotemustine	DAB	PR	147
7	F	82	M1c	H	Dacarbazine	VEMU	PD	-
8	M	81	M1c	H	None	VEMU	PD	-
9	M	57	M1c	H	None	VEMU	PR	223
10	M	60	M1b	N	None	DAB+TRAM	PR	1082
11	M	45	M1c	H	None	DAB+TRAM	PR	188
12	M	70	M1c	N	None	DAB+TRAM	PR	117
13	M	65	M1c	H	None	VEMU	PD	-
14	M	49	M1c	H	None	VEMU	PR	147
15	M	39	M1b	H	None	DAB	PD	-
16	M	81	M1c	H	None	VEMU	PD	-
17	F	46	M1c	H	None	DAB+TRAM	PR	244
18	M	56	M1c	N	Nivolumab	DAB+TRAM	PR	504
19	M	57	M1c	H	None	DAB+TRAM	PR	172
20	M	35	M1c	H	None	DAB+TRAM	PR	180
21	F	78	M1a	N	None	DAB+TRAM	SD	83
22	M	66	M1b	N	None	DAB+TRAM	CR	1387
23	M	61	M1c	N	None	DAB+TRAM	PR	333
24	F	67	M1c	H	None	VEMU+COBI	PR	270
25	M	47	M1a	N	None	DAB+TRAM	PD	-
26	F	49	M1c	N	None	DAB+TRAM	PR	476

^a^Stage at first serum collection (i.e. T0).

^b^N, normal; H, >1.5 upper limit of normal values

^c^DAB, dabrafenib. TRAM, trametinib. VEMU, vemurafenib, COBI, cobimetinib. In parenthesis, months of monotherapy before trametinib addiction.

^d^Best response according to RECIST 1.1 criteria: PR, partial response; SD, stable disease; PD, progressive disease.

^e^TTF, time to treatment failure.

VEGF-A levels were undetectable in 7 out of the total serum samples tested (T0, n=2; T2, n=3; TP, n=2).

**Fig. 11:**
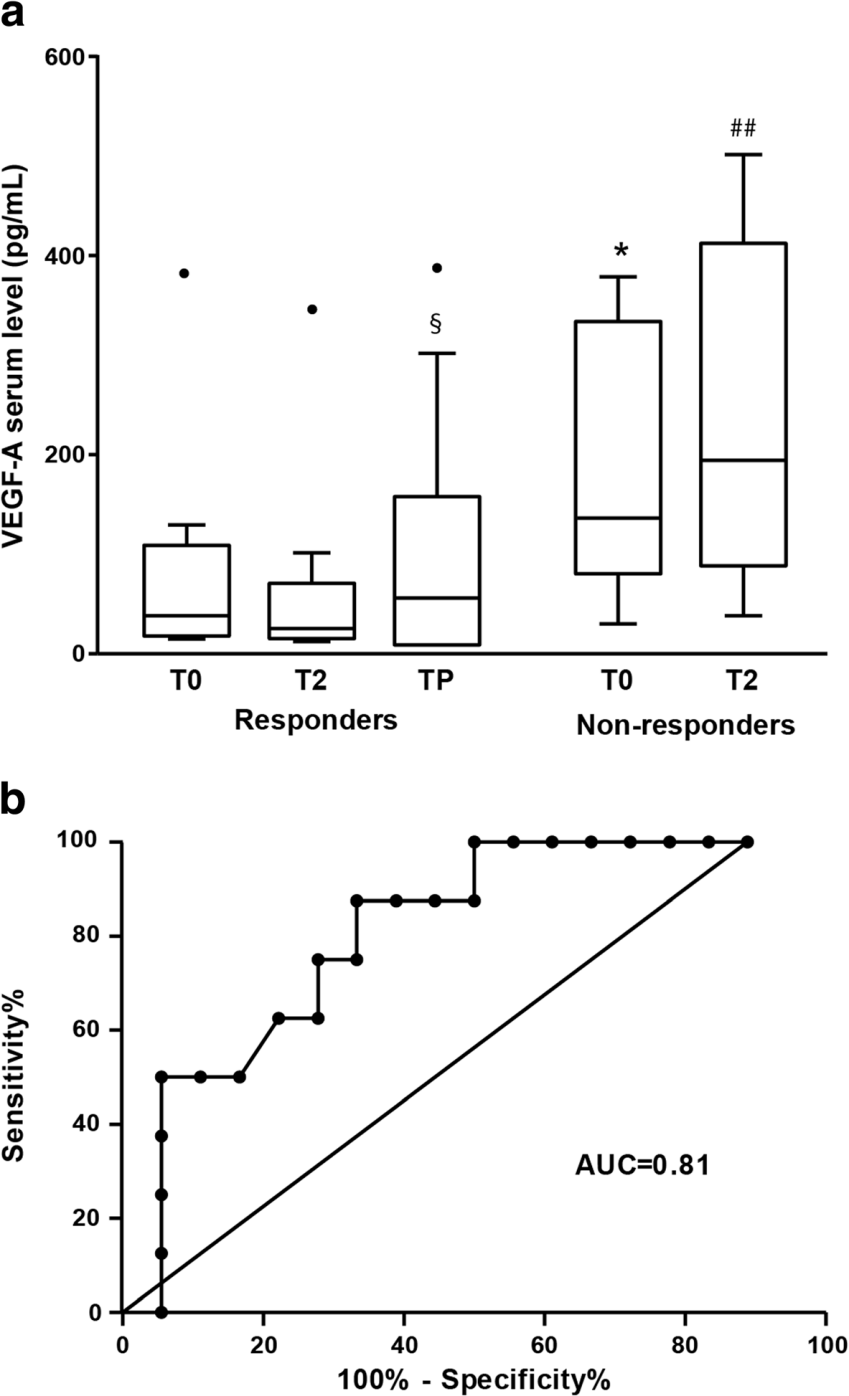
**a** Box-and-whisker diagrams of VEGF-A serum levels in melanoma patients treated with BRAFi or BRAFi+MEKi. VEGF-A serum levels were measured in 26 melanoma patients (18 responders and 8 non-responders) before the start of therapy (T0), after two months of treatment (T2) and at disease progression (TP). The edges of each box represent the 75th and 25th percentile, respectively, and whiskers the maxima and minima. The horizontal bar within each box indicates the median. The outliers are denoted by dots. Data were analyzed by non-parametric Wilcoxon matched-pairs signed-rank test. ^*^P<0.05 T0 non-responders *vs* T0 responders; ^§^*P*<0.05 TP responders *vs* T2 responders; ^##^*P*<0.01 T2 non-responders *vs* T2 responders. **b** Receiver operating characteristics curves (ROC) of VEGF-A levels in responder versus non-responder patients.

Fig. 11 shows the results of statistical analyses performed on VEGF-A serum levels detected at the different time points in all responder and non-responder patients.

At baseline (T0) and at T2, statistically significant differences were observed in VEGF-A levels between the responder and the non-responder group. The median VEGF-A levels at baseline was 25.5 pg/ml (IQR=7.7, 95.3) for the responders and 128.8 pg/ml (IQR=77.7, 319.3) for non-responders (Mann-Whitney U test, *P*=0.013), whereas the median VEGF-A levels at T2 was 14.1 pg/ml (IQR=1.96, 60.7) for the responders and 188.3 pg/ml (IQR=103.5, 336.9), for non-responders (Mann-Whitney U test, *P*=0.002).

Compared to the baseline, a decrease in VEGF-A level was observed at T2 in responders, with an overall mean decrease of -12.6 pg/ml (95% CI= - 24.5, -0.70), with a clear tendency to a significant difference in the before-after comparison (Wilcoxon matched-pairs signed-rank test, *P*=0.055). Furthermore, a statistically significant increase of VEGF-A levels was observed at TP in comparison with T2, with a median level 49.1 pg/ml (IQR=0.48, 148.0) and with an overall mean increase of 44.8 pg/ml (95% CI= 9.66, 80.0; Wilcoxon matched-pairs signed-rank test, *P*=0.037).

In non-responder patients, the VEGF-A levels at T2 were similar to the baseline levels, with a median level of 188.3 pg/ml (IQR=103.5, 336.9; Wilcoxon matched-pairs signed-rank test, *P*=0.779).

Serum levels of VEGF-A detected in each patient at the different time points are reported in Additional file 4: Figure S4.

Serum level of VEGF-A at T0 was used to construct ROC curve in order to estimate its potential as biomarker predictive of patient’s response to therapy. The Area Under Curve (AUC) of VEGF-A levels in responders versus non-responders was 0.81, with 95% CI from 0.64 to 0.98 (Fig. 11b). The cut-off value of VEGF level with the maximum sum of sensitivity and specificity was 60 pg/ml, with a sensitivity of 87.5% and a specificity of 66.7%.

## Discussion

A major challenge for targeted therapy in BRAF-mutant melanoma is the development of secondary resistance. Although most of the resistance mechanisms identified so far involve genomic alterations, increasing experimental evidence points out that epigenetic and/or post-transcriptional mechanisms can play a relevant role in the emergence of drug resistance. In this context, some recent investigations on sensitive and matched BRAFi-resistant cell lines demonstrated changes of miRNA expression pattern in the latter cell lines, and implicated selected miRNAs in the regulation of cell responses to BRAFi [18–25].

In this study, we further explored the role of miRNAs in melanoma acquired resistance to BRAFi. Comparing miRNA expression profiles of a BRAFi-sensitive cell line (i.e. A375) and its dabrafenib-resistant subline (i.e. A375R), we identified 27 miRNAs whose expression was significantly altered in the latter cell line. Those miRNAs can be involved in acquired drug resistance and/or in some biological characteristics of the resistant cells we had previously identified, namely enhanced invasive capacity, increased secretion of VEGF-A and MMP-9, and ERK1/2 and AKT hyperactivation [27, 28]. Indeed, annotations of their target genes for the enrichment of KEGG pathways identified a number of pathways already implicated in the regulation of cell proliferation and invasiveness and/or resistance to BRAFi, including MAPK- and PI3K/AKT-signaling pathways.

Among miRNAs identified as being differentially expressed between sensitive- and dabrafenib-resistant cells, we focused our attention on miR-126-3p, markedly down-regulated in the resistant cells. miR-126-3p (also referred to as miR-126) and its complement miR-126-5p (also referred to as miR-126* or, previously, miR-123) derived from the same *miR* gene located at chromosome 9q24.3, within intron 7 of the epidermal growth factor-like domain 7 (*EGFL7*) gene [42]. A large number of experimental studies have demonstrated that miR-126-3p, beside regulating angiogenesis and vessel integrity, acts as a tumor suppressor in a variety of tumors, including melanoma, through negative modulation of proliferation, migration and invasion [26, 42, 56]. Moreover, Lin et al [57] demonstrated a progressive decrease of miR-126-3p levels in dysplastic nevi, primary melanomas and melanoma metastases and a shorter 5-years overall survival in patients with primary tumor expressing low miR-126-3p levels. Down-regulation of miR-126-3p has also been implicated in acquired resistance of cervical cancer cells to TRAIL [58], and of gastric cancer cells to vincristine and adriamycin [59]. Furthermore, enforced expression of miR-126-3p in drug treatment-naive non-small cell lung cancer cells [42], renal cancer cells [60] or breast cancer cells [61] has been shown to increase sensitivity to vincristine and adriamycin, cisplatin, and CDK4/6 or PIK3CA inhibitors, respectively. On the other hand, no data are available on the role of miR-126-3p&5p in melanoma drug resistance.

In the present study, we show for the first time that miR-126-3p was down-regulated in melanoma cells with acquired resistance to the BRAFi dabrafenib and that restoration of miR-126-3p expression in those cells promoted cell cycle arrest at the G0/G1 phase, reduced the levels of phosphorylated ERK1/2 and/or AKT, and caused a significant inhibition of proliferation and invasiveness. Importantly, miR-126-3p replacement in the resistant cells also affected their response to dabrafenib. Specifically, restoration of miR-126-3p expression significantly increased dabrafenib sensitivity in A375R cells and abrogated the growth promoting effect of the drug in SK-Mel28R cells. Moreover, in A375R cells, miR-126-3p replacement counteracted the stimulating effect of dabrafenib on the invasive capacity. In agreement with a functional role of miR-126-3p down-regulation in acquired resistance to BRAFi, we found that dabrafenib treatment induced up-regulation of miR-126-3p in A375 and SK-Mel28 cells but not in their drug-resistant counterparts, and that enforced expression of that miR in the sensitive cells impaired proliferation and delayed the emergence of secondary resistance upon chronic exposure to the drug. Taken together, our data suggest that in patients with BRAF-mutant melanoma, the combination of BRAFi with miR-126-3p mimics could represent a novel therapeutic approach to counteract the development of resistance as well as to impair growth and metastasis of tumors become unresponsive to BRAFi. Although there are still several challenges in miRNA replacement therapy, the promising results obtained in two phase 1 clinical trials recently concluded in patients with advanced solid tumors [62] and pleural mesothelioma [63] highlight the potential of this approach.

In previous studies, the tumor suppressor and/or the chemosensitizing activity of miR-126-3p has been linked to down-modulation of different target genes, among which are, for instance, *VEGFA* and *PIK3R2* in non-small cell lung cancer and breast cancer [42], *IRS1* in breast cancer [42] *EZH2* in gastric cancer [59] *SLC7A5* in renal carcinoma [60], *CFLAR/c-FLIP* in cervical cancer [58], *CXCR4* in colon and thyroid cancer [42, 46]. Up to date, only *ADAM9*, *MMP7* and *PIK3R2* have been validated as direct miR-126-3p&5p target genes in drug treatment-naïve melanoma cells [26]. In this report, we showed that miR-126-3p was able to down-regulate ADAM9 protein levels also in dabrafenib-resistant melanoma cells, and that this molecular event was involved in the biological effects produced in the resistant cells by restoration of miR-126-3p expression. Indeed, *ADAM9* silencing caused a marked inhibition of cell proliferation and invasiveness. Moreover, it increased dabrafenib sensitivity in A375R cells and counteracted the growth promoting effects of the drug in SK-Mel28R cells. Importantly, in dabrafenib-sensitive A375 and SK-Mel28 cells, continuous down-regulation of ADAM9 levels by specific siRNAs, markedly delayed the onset of resistance upon prolonged exposure to the drug. Targeting ADAM9 may be, therefore, an effective strategy to increase the efficacy of BRAFi.

In the present investigation, we also demonstrated that VEGF-A secretion was significantly reduced in A375R and SK-Mel28R cells transfected with pre-miR-126-3p, and using a luciferase assay we validated VEGF-A as a direct target of miR-126-3p in dabrafenib-resistant cells. These results are in agreement with the findings that both A375R and SK-Mel28R cells express lower levels of miR-126-3p and, as previously reported [27, 28], secrete higher amount of VEGF-A as compared with their dabrafenib-sensitive counterparts. Down-regulation of VEGF-A, in addition to that of ADAM9, appears to be involved in the biological effects exerted by miR-126-3p replacement in the drug resistant cells. Indeed, VEGF-A silencing almost abrogated proliferation and invasiveness of A375R cells. Of note, inhibition of cell growth and ECM invasion induced in the resistant cells by miR-126-3p replacement was lower than that caused by direct VEGF-A silencing. This can be explained taking into account that the anti-VEGF-A siRNA was more effective than miR-126-3p replacement in reducing VEGF-A expression. While this paper was in preparation, Fattore et al [21] demonstrated up-regulation of VEGF-A in melanoma cell lines with acquired resistance to vemurafenib and identified miR-199b-5p as a negative regulator of that cytokine. miRNA expression profiling of A375 and A375R did not reveal a differential expression of miR-199b-5p. It appears, therefore, that activation of the VEGF-A signaling pathway is a common feature to which melanoma cells can converge during development of resistance to BRAFi, and that this can be accomplished through down-modulation of different VEGF-A targeting miRs. A recent study conducted on BRAF-mutant melanoma xenografts [64] demonstrated that the combination of the BRAFi PLX4720 and bevacizumab was more effective than each single agent in inhibiting tumor growth and metastasis and also delayed the onset of acquired resistance. Since bevacizumab does not bind to murine VEGF-A, the study highlights the importance of neutralizing VEGF-A secreted by melanoma cells to increase BRAFi efficacy. On the other hand, it has been demonstrated that VEGF-A, in addition to paracrine and autocrine signals, can also mediate intracrine signaling loops promoting proliferation, survival, invasiveness and chemoresistance of tumor cells [65–68]. It can be, therefore, hypothesized that a better antitumor activity and a greater delay in the onset of secondary resistance could be achieved by combining BRAFi with strategies inhibiting VEGF-A expression in cancer cells.

In the last few years, increasing experimental evidence has been accumulated showing that the “liquid biopsy” represented by peripheral blood can be a useful biological sample to identify, with a non-invasive modality, biomarkers (i.e. DNA, RNA, miRNAs, proteins) to predict and monitor response to therapy, and to analyze tumor evolution under the treatment pressure [69, 70]. Up to date, only a few studies have addressed the role of circulating cytokine/chemokine/growth factors as potential biomarkers of patients’ response to BRAFi or BRAF+MEKi therapy. In particular, Wilmott et al [71], performed a serum cytokine profiling in a cohort of 24 patients treated with dabrafenib, vemurafenib or dabrafenib+trametinib and found that high pre-treatment levels of CCL2 were associated with poor clinical response. Accordingly, Vergani et al [22] , in a cohort of 33 patients treated with vemurafenib found that pre-therapy levels of CCL2 were higher in patients whose clinical response lasted ≤6 months than in patients with a response enduring at least two years. More recently, we reported that a decrease of plasmatic PTTG1 protein could be detected early on-treatment in about 70% of patients responding to BRAFi or BRAF+MEKi and in only 20% of non-responding patients [27]. In the present study, we showed that pre-treatment (T0) and early on-treatment (T2) levels of serum VEGF-A were significantly higher in patients who did not respond to therapy than in patients who achieved CR or PR. Furthermore, using VEGF-A serum level at T0 as a biomarker, it was possible to discriminate therapy responding from non-responding patients with a good sensitivity and specificity [72]. Although these results need to be validated in a larger cohort of patients, the level of circulating VEGF-A appears to be an easily measurable biomarker with potential clinical utility to predict and/or monitor patient’s response to therapy with BRAFi or BRAFi+MEKi. Noteworthy, elevated serum levels of VEGF-A were previously shown to be associated with poor clinical response and decreased overall survival in melanoma patients treated with high-dose interleukin-2 [54] or with ipilimumab [55]. Moreover, in pre-clinical models of melanoma, host immunity was found to contribute to the antitumor activity of BRAFi [73]. The immunosuppressive activity of VEGF-A, in addition to the direct effects of that cytokine on tumor vasculature and melanoma growth and metastasis, might therefore underlie the association of high serum levels of VEGF-A with a poor clinical response to BRAFi and MEKi.

## Conclusions

In conclusion, our study confirms the tumor suppressor role of miR-126-3p in melanoma and demonstrates that its down-regulation is involved in acquired resistance to dabrafenib. Replacement of miR-126-3p or inhibition of its targets VEGF-A or ADAM9 appear valuable strategies to impair proliferation and invasiveness of dabrafenib-resistant cells and to increase their drug sensitivity. Finally, we show for the first time that serum level of VEGF-A may represent a promising biomarker for predicting patients’ response to BRAFi or BRAFi+MEKi and for monitoring the onset of resistance.

## Additional files

Additional file 1: **Figure S1.** MMP7 and CXCR4 expression in A375 and SK-Mel28 cell lines and their dabrafenib-resistant sublines. Melanoma cell lysates were analyzed by immunoblotting using antibodies against MMP7 and CXCR4, or against β-tubulin as a loading control. The results are representative of two independent experiments. (TIF 132 kb)

Additional file 2: **Figure S2.** Dabrafenib treatment down-regulates ADAM9 expression in A375 and SK-Mel28 cells. Melanoma cells were incubated with 100 nM dabrafenib (+) or with DMSO alone (-) and after 48 h ADAM9 expression was evaluated by immunoblotting. Antibody against β-tubulin was used as a loading control. The results are representative of two independent experiments. (TIF 70 kb)

Additional file 3: **Figure S3.** *ADAM9* silencing delays the development of resistance to dabrafenib. **a** SK-Mel28 cells were seeded into 96-well plates and every eight days transfected with 10 nM siADAM9 or siCTRL and treated with 100 nM dabrafenib (DAB) or DMSO. On day 0 (i.e after the first transfection), 8, 24 and 32, the cells were fixed, stained with crystal violet and photographed before quantitative analysis of proliferation. Images from a representative experiment are shown. **b** Quantitative analysis of proliferation of cell cultures described in (a). Crystal violet was solubilized and absorbance was read at 595 nm. Each value represents the arithmetic mean of three independent experiments performed with triplicate cultures. Bars, SEM. ^**^P<0.01, siADAM9 *vs* matched siCTRL; ^§§^*P*<0.01, siCTRL/DAB/Day 8 *vs* siCTRL/DMSO/Day 8; ^††^*P*<0.01, siADAM9/DAB/Day 8 *vs* siADAM9/DMSO/Day8; ^##^*P*<0.01, siCTRL/DAB/Day 24 *vs* siCTRL/DAB/Day 8; ^¶^*P*<0.05, siCTRL/DAB/Day 32 *vs* siCTRL/DAB/Day 24. (TIF 354 kb)

Additional file 4: **Figure S4.** Quantification of VEGF-A in serum of melanoma patients treated with BRAFi or BRAFi+MEKi. VEGF-A levels were determined by ELISA in serum samples of 18 responder (**a**) and 8 non-responder (**b**) melanoma patients before the start of therapy (T0), after two months of treatment (T2) and at disease progression (TP). One patient among responders (case #11) displayed undetectable VEGF-A serum levels at all time points analyzed and was not included in the figure. Each value represents the arithmetic mean ± SEM of two independent determinations. (TIF 164 kb)

## Data Availability

The datasets generated and analyzed during the current study are available in the Gene Expression Omnibus repository: https://www.ncbi.nlm.nih.gov/geo/query/acc.cgi?acc=GSE117666

## Abbreviations

ADAM9: A disintegrin and a metalloproteinase domain 9
BRAFi: BRAF inhibitors
CM: Complete Medium
CR: Complete Response
CT: Computer tomography
C_T_: Threshold Cycle
CXCL12: C-X-C motif chemokine ligand 12
CXCR4: C-X-C motif chemokine receptor 4
DMSO: Dimethyl Sulfoxide
ECM: Extracellular Matrix
EGFL7: Epidermal growth factor-like domain 7
EGFR: Epidermal Growth Factor Receptor
FDR: False Discovery Rate
IGF-1R: Insulin-like Growth Factor-1 Receptor
IQR: Interquartile Range
mAb: Monoclonal Antibody
MEKi: MEK inhibitor
miRNAs: microRNAs
MMP7: Matrix metallopeptidase 7
MTT: 3-(4,5-dimethylthiazol-2-yl)-2,5-diphenyltetrazolium bromide
PBS: Phosphate-Buffered Saline
PD: Progressive Disease
PR: Partial Response
qRT-PCR: Real-time quantitative RT-PCR
RTKs: Receptor Tyrosine Kinases
SAM: Significance Analysis of Microarrays
SD: Stable Disease
SDS: Sodium Dodecyl Sulphate
SEM: Standard Error of the Mean
TBST: Tris-Buffered Saline supplemented with 0.1% Tween 20
TTF: Time to Treatment Failure
