# Supplementary figures and images for: miR-126-3p down-regulation contributes to dabrafenib acquired resistance in melanoma by up-regulating ADAM9 and VEGF-A
Source: J Exp Clin Cancer Res. 2019 Jun 21;38:272. doi: 10.1186/s13046-019-1238-4 (PMC6588909; doi:10.1186/s13046-019-1238-4)

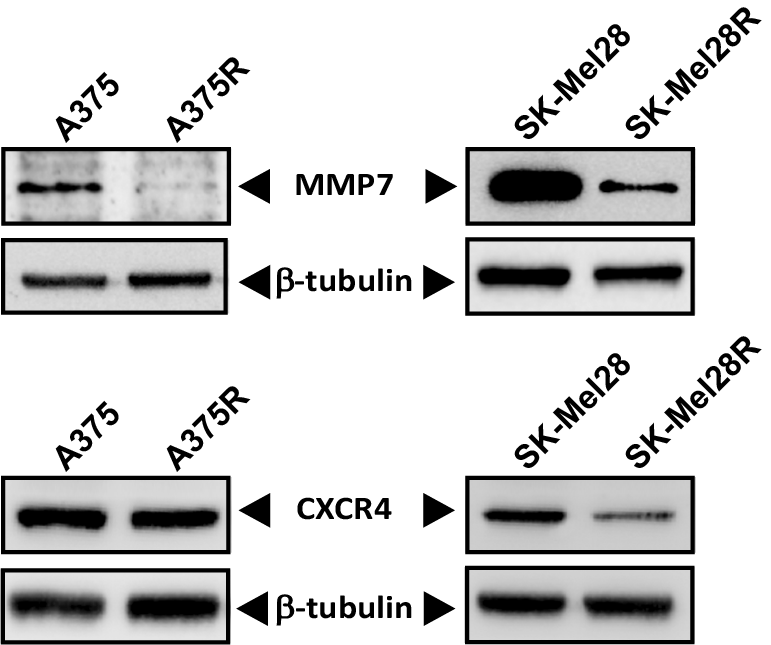

Supplement: Supplementary file 1 — Figure S1. MMP7 and CXCR4 expression in A375 and SK-Mel28 cell lines and their dabrafenib-resistant sublines. Melanoma cell lysates were analyzed by immunoblotting using antibodies against MMP7 and CXCR4, or against β-tubulin as a loading control. The results are representative of two independent experiments. (TIF 132 kb) [file 13046_2019_1238_MOESM1_ESM.tif]

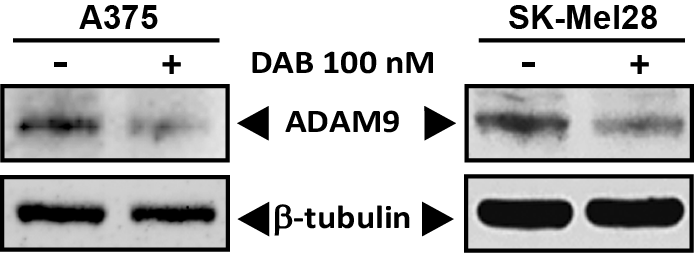

Supplement: Supplementary file 2 — Figure S2. Dabrafenib treatment down-regulates ADAM9 expression in A375 and SK-Mel28 cells. Melanoma cells were incubated with 100 nM dabrafenib (+) or with DMSO alone (-) and after 48 h ADAM9 expression was evaluated by immunoblotting. Antibody against β-tubulin was used as a loading control. The results are representative of two independent experiments. (TIF 70 kb) [file 13046_2019_1238_MOESM2_ESM.tif]

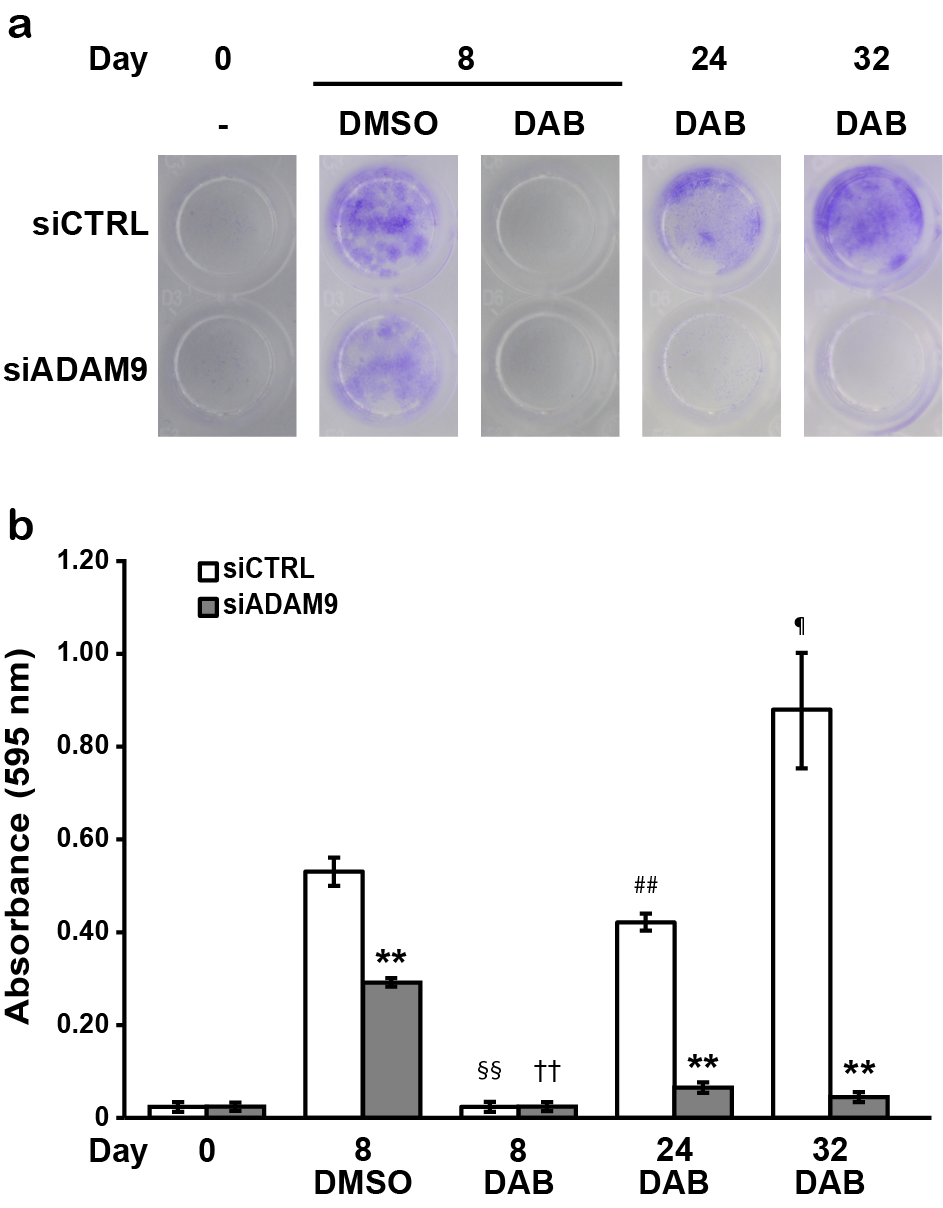

Supplement: Supplementary file 3 — Figure S3. ADAM9 silencing delays the development of resistance to dabrafenib. a SK-Mel28 cells were seeded into 96-well plates and every eight days transfected with 10 nM siADAM9 or siCTRL and treated with 100 nM dabrafenib (DAB) or DMSO. On day 0 (i.e after the first transfection), 8, 24 and 32, the cells were fixed, stained with crystal violet and photographed before quantitative analysis of proliferation. Images from a representative experiment are shown. b Quantitative analysis of proliferation of cell cultures described in (a). Crystal violet was solubilized and absorbance was read at 595 nm. Each value represents the arithmetic mean of three independent experiments performed with triplicate cultures. Bars, SEM. **P<0.01, siADAM9 vs matched siCTRL; §§P<0.01, siCTRL/DAB/Day 8 vs siCTRL/DMSO/Day 8; ††P<0.01, siADAM9/DAB/Day 8 vs siADAM9/DMSO/Day8; ##P<0.01, siCTRL/DAB/Day 24 vs siCTRL/DAB/Day 8; ¶P<0.05, siCTRL/DAB/Day 32 vs siCTRL/DAB/Day 24. (TIF 354 kb) [file 13046_2019_1238_MOESM3_ESM.tif]

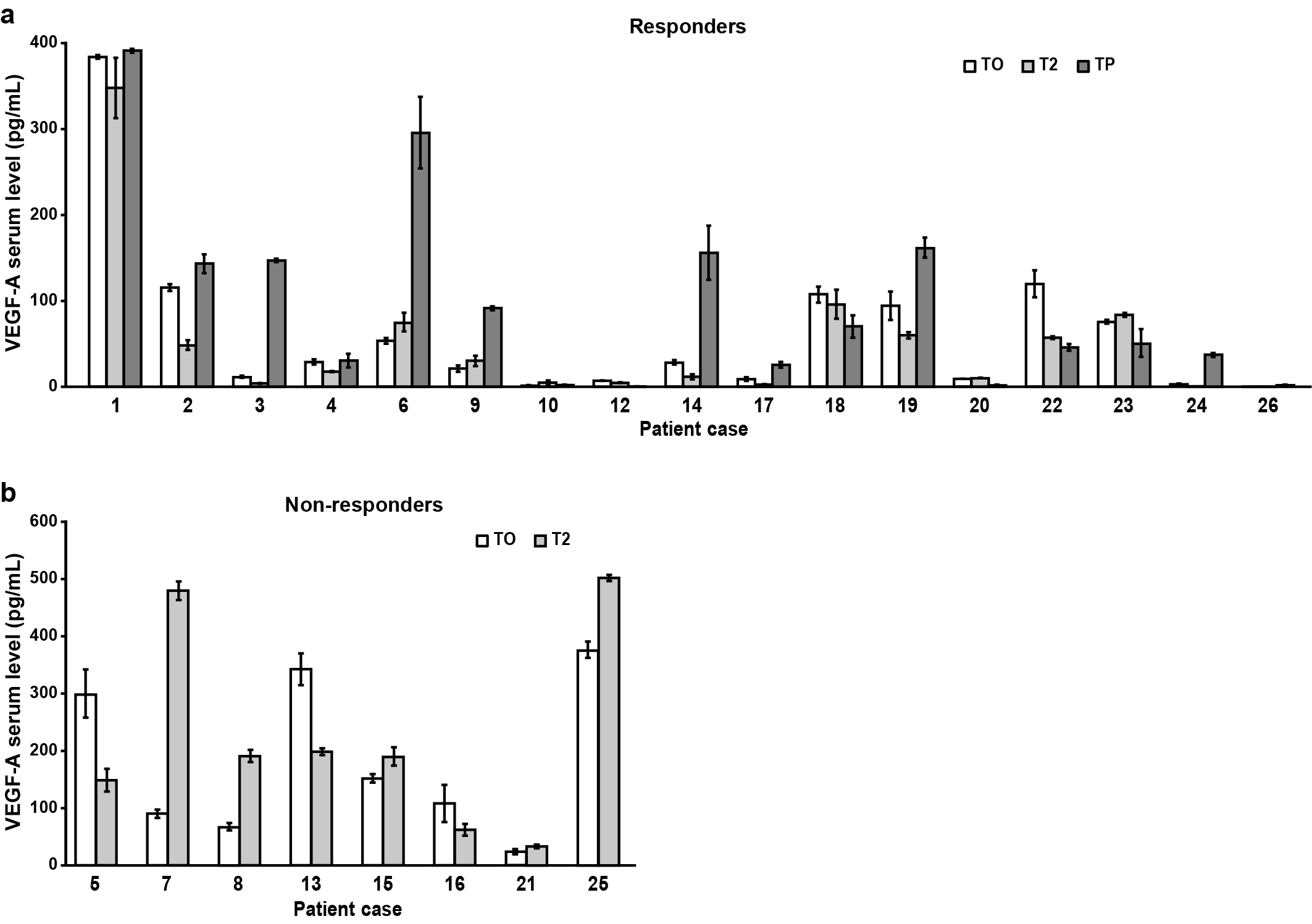

Supplement: Supplementary file 4 — Figure S4. Quantification of VEGF-A in serum of melanoma patients treated with BRAFi or BRAFi+MEKi. VEGF-A levels were determined by ELISA in serum samples of 18 responder (a) and 8 non-responder (b) melanoma patients before the start of therapy (T0), after two months of treatment (T2) and at disease progression (TP). One patient among responders (case #11) displayed undetectable VEGF-A serum levels at all time points analyzed and was not included in the figure. Each value represents the arithmetic mean ± SEM of two independent determinations. (TIF 164 kb) [file 13046_2019_1238_MOESM4_ESM.tif]
